# Machine learning with optimization to create medicine intake schedules for Parkinson’s disease patients

**DOI:** 10.1371/journal.pone.0293123

**Published:** 2023-10-18

**Authors:** Tomasz Gutowski, Ryszard Antkiewicz, Stanisław Szlufik

**Affiliations:** 1 Military University of Technology, Warsaw, Poland; 2 Medical University of Warsaw, Warsaw, Poland; Univerzitet Singidunum, SERBIA

## Abstract

This paper presents a solution for creating individualized medicine intake schedules for Parkinson’s disease patients. Dosing medicine in Parkinson’s disease is a difficult and a time-consuming task and wrongly assigned therapy affects patient’s quality of life making the disease more uncomfortable. The method presented in this paper may decrease errors in therapy and time required to establish a suitable medicine intake schedule by using objective measures to predict patient’s response to medication. Firstly, it demonstrates the use of machine learning models to predict the patient’s medicine response based on their state evaluation acquired during examination with biomedical sensors. Two architectures, a multilayer perceptron and a deep neural network with LSTM cells are proposed to evaluate the patient’s future state based on their past condition and medication history, with the best patient-specific models achieving R^2^ value exceeding 0.96. These models serve as a foundation for conventional optimization, specifically genetic algorithm and differential evolution. These methods are applied to find optimal medicine intake schedules for patient’s daily routine, resulting in a 7% reduction in the objective function value compared to existing approaches. To achieve this goal and be able to adapt the schedule during the day, reinforcement learning is also utilized. An agent is trained to suggest medicine doses that maintain the patient in an optimal state. The conducted experiments demonstrate that machine learning models can effectively model a patient’s response to medication and both optimization approaches prove capable of finding optimal medicine schedules for patients. With further training on larger datasets from real patients the method has the potential to significantly improve the treatment of Parkinson’s disease.

## Introduction

The objective of this paper is to present a new method for determining drug dosing in people with Parkinson’s disease (PD). The method should ensure that periods and severity of disease symptoms are minimized. It should also minimize the adverse effects of excessive drug doses. PD is a chronic and progressive neurodegenerative disorder that affects millions of people worldwide. It is characterized by the loss of dopaminergic neurons in the brain, leading to motor symptom manifestations such as tremors, rigidity, and bradykinesia, as well as non-motor symptoms such as cognitive impairment, depression and sleep disturbances [1]. The management of PD is complex and requires a multidisciplinary approach, including pharmacological and non-pharmacological interventions.

Levodopa, a dopamine precursor, is the most effective drug for treating the motor symptoms of PD. However, levodopa therapy is associated with several challenges, including the development of motor fluctuations and dyskinesia, which can significantly impact patients’ quality of life [2, 3]. To minimize these adverse effects and optimize treatment outcomes, it is essential to individualize levodopa dosing based on each patient’s unique symptom profile and response to medication.

Traditionally, levodopa dosing has been based on clinical assessments conducted by healthcare professionals, which are typically conducted every few months. However, these traditional methods have significant limitations, including infrequent assessments and subjective measures that can impact the quality of life for patients. These assessments are usually performed using questionnaires and scales to objectively evaluate the patient’s current condition and their response to the medication such as the MDS-UPDRS [4], patient diaries [5], Hoehn and Yahr Scale [6] etc. However, these assessments may not accurately capture the full range of symptoms that patients experience and may not reflect the variability in symptoms that can occur throughout the day. Moreover, these assessments are often subject to inter- and intra-rater variability, leading to inconsistent dosing recommendations. The low frequency of visits also makes it very difficult for the clinician to receive continuous objective feedback and apply adjustments to the medication schedule. This highlights the need for a more dynamic, real-time approach to levodopa dosing.

Taking medicine following an unsuitable, not personalized medicine schedule, might lead to a low quality of life for the patient. Patients may struggle to perform daily tasks and experience emotional distress due to their condition—experiencing severe PD symptoms. Additionally, they may experience severe dyskinesia if the concentration of levodopa is too high. Patients may experience negative effects from either or both of these conditions if the medication intake schedule is not personalized to their needs.

From the above description, the problem of the correct dosage of medication for people with PD can be decomposed into three problems:

- assessment of the patient’s current state, derived from the intensity of PD symptoms (tremor, bradykinesia, muscle stiffness, dyskinesias, etc.)
- predicting the patient’s response over time to the medication doses used, assuming that the patient’s current state and the timing and size of previous medication doses are known;
- optimizing the medication schedule.

To address the first problem, recent studies have investigated the possibilities to objectively evaluate PD symptoms [7–11]. This has been commonly performed using a set of biomedical sensors worn by the patients and mobile devices. The sensors collected data while the patient completed specific tasks or continuously, in the background, during daily activities. The collected data was then used to assess the patient’s condition. Two approaches have been taken to compute the evaluation. The first approach involves heuristically designed algorithms that calculate values indicating the patient’s condition using signal data [7, 9]. The second approach is to use machine learning, which has achieved great results in recent years. Machine learning techniques, such as Convolutional Neural Networks and LSTM [12], have been extensively studied for medical imaging and time-series forecasting applications, including brain tumor classification [13], COVID-19 diagnostics [14, 15], and healthcare analysis [16]. Research has focused on predicting specific symptom severities such as tremor, bradykinesia, and dyskinesia [8, 10, 11, 17] as well as providing an overall score for patient’s condition using previously defined scales [18–23]. The prediction of a score representing the overall patient’s condition may give the best results when monitoring and evaluating the current therapy. In [22], sensor signals (collected during the pronation-supination task) have been used to predict the outcome of a Treatment Response Scale (TRS). It represents the patient’s response to the taken medication with values between -3 (severe symptoms) and 3 (very dyskinetic) with 0 being the optimal state. Monitoring of TRS values throughout the day can greatly assist the clinicians in adjusting the treatment.

Understanding the pharmacokinetics and pharmacodynamics of levodopa [24] is crucial for defining medicine schedules in PD. Recent studies have presented PK/PD models [25] that consider the pharmacokinetics and pharmacodynamics of duodenal levodopa infusion [26, 27]. These models allow for estimating the concentration of levodopa in each of the compartments and provide an equation that can be used to calculate the TRS result based on the concentration in the effect compartment. In [28] the model was utilized to optimize the levodopa infusion rate.

The problem of optimizing drug dosing for PD patients can be divided into two subproblems: continuous infusion dosing and oral dosing. Optimizing the oral administration of levodopa is a more complex task, due to the irregular release of the medication into the bloodstream. However, attempts to face this challenge have already been made. One of the articles [29] uses the previously mentioned PK/PD models for optimizing the oral administration of levodopa/carbidopa microtablets. During the study the patients were equipped with sensors and the collected sensor data was used to predict the TRS values. These were then used to fit individual (for every patient) PK/PD dose response models, to find the best medicine schedule in the considered solution space. The authors considered a morning dose and equal maintenance doses taken at regular intervals. After generating all possible solutions, the one with the smallest area outside the target (optimal) range for TRS was selected.

The approach presented in [30] utilizes Deep Reinforcement Learning to determine the timing and dosage of medication. The authors used the clinical literature to create hypothetical patient profiles and their sample sensor response for two symptoms: bradykinesia and dyskinesia after medication administration. The A3C algorithm was used by the agents to learn the policy for dosing levodopa/carbidopa medication.

In this paper a novel approach is presented, it aims to individualize PD oral medicine intake schedules for every patient. Specifically, this study contributes by introducing a unique algorithm for personalized medication schedules, utilizing machine learning to predict patient responses and thereby increase the precision of dosing recommendations. The designed algorithm has three fundamental steps. Firstly, collect data from patients regarding their medication intake and their response to the medication (how that patient’s state changes during the day, how the medication impacts it). Secondly, train a machine learning model to predict the patient’s response to the medication. Lastly, utilize the model to find a schedule that optimizes therapy (minimizes disease symptoms and reduces the occurrence of side effects such as levodopa induced dyskinesias). Our hypothesis is that collecting objective and frequent data on patients’ symptom profiles would allow for more precise dosing recommendations, leading to better symptom control, improved quality of life and reduced waiting time to achieve satisfactory medicine schedules. This paper makes several contributions to the field: (1) it introduces a new method for individualizing PD medicine schedules using machine learning, (2) it integrates sensor data for more accurate patient profiling, (3) it uses both conventional optimization and reinforcement learning techniques for medication scheduling, (4) it combines the patient’s response to medication model with creating medicine schedules in one framework.

To find the optimal medicine intake schedule we have employed two approaches. The first one uses conventional optimization algorithms to find the schedule that minimizes the value of the objective function, which can represent the difference between the patient’s real state and the optimal state or can result in minimizing the occurrences when the patient’s state falls out of the target range. The second approach uses reinforcement algorithms enabling the agents to learn the optimal policy, the maintains the patient in the desired state throughout the day.

The presented study focuses on finding the medicine intake schedule for levodopa/carbidopa pills. However, with slight modification it could be applied to other PD related medication and optimize the intake of multiple substances such as levodopa and dopamine agonists. To avoid the constraining of the solution to just levodopa/carbidopa pills, it will be referred to as PD medicine.

For the purpose of this paper, data of 50 simulated PD patients was analyzed. The data for each patient included TRS scores, which represent the patient’s state (e.g., the output of an ML model based on sensor data input during pronation-supination task [22]). These scores were generated with 10-minute intervals over a period of 3 days (excluding sleep time) using PK/PD models, that were primarily developed for duodenal levodopa infusion, but were later modified to model oral medicine intake [23]. These patients were generated along with levodopa/carbidopa pills doses which influenced their condition throughout the day.

The data generated with PK/PD models was analyzed and machine learning algorithms were employed to capture the character of the patient specific medicine response as well as identify patterns in symptom fluctuation. However, if trained on real patient data, the models can capture individual features not considered in the definition of the defined PK/PD model such as physical activity and eating patterns. Using ML models not only provides greater individualization but also simplifies the procedures required for treatment customization, making it more scalable and applicable in real-world situations. Patients are only asked to complete a few exercises during a short period when they take the medication, allowing the model to adapt to their unique response.

After training the individual medicine response models the process of finding the best medicine schedule begins using two of the described approaches (optimization using heuristic algorithms and reinforcement learning (RL)). Both approaches utilize patient specific dose response models.

The remainder of this paper consists of 4 parts. The *Materials and Methods* section starts with the description of the used dataset of PD patients. It contains the description of the machine learning models used for patient state prediction along with the explanation of the optimization approaches used for creating medicine schedules. It is followed by the *Results* section which is based on tables presenting the results of used machine learning models, their metrics along with medicine intake schedules created through optimization. The *Discussions* section presents an overview of the achieved results, the practical implications of using machine learning models for therapy assignments in Parkinson’s disease are also discussed, including the limitations and potential biases in the research. This final chapter—*Conclusions* summarizes the key findings of the research and discusses its broader impact.

## Materials and methods

**Fig 1:**
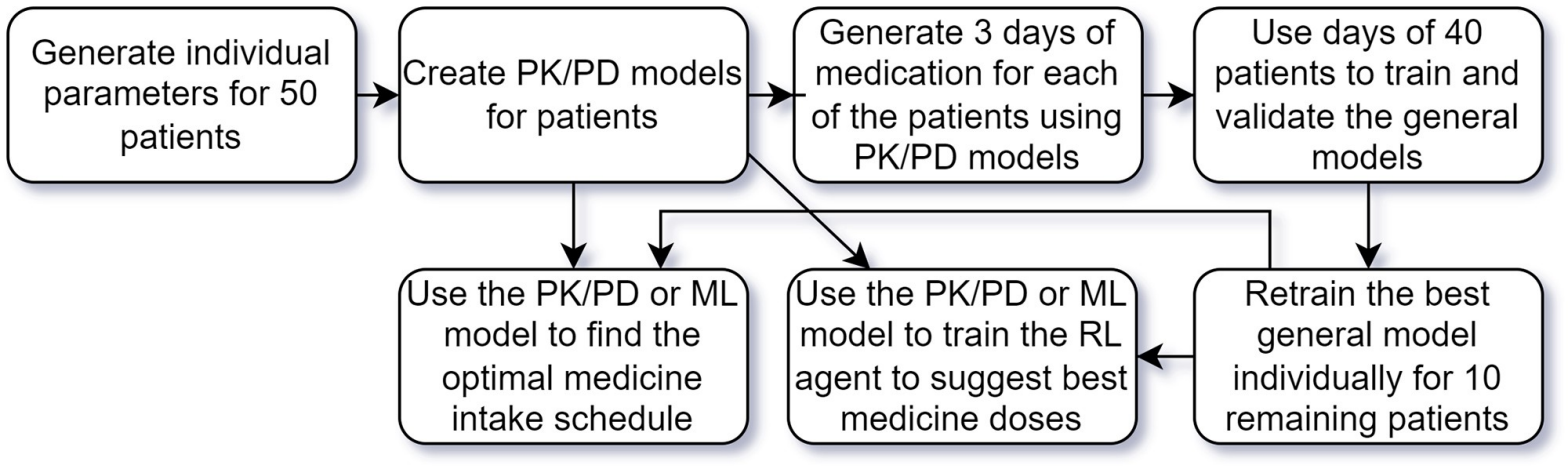
Diagram representing the steps in the presented method to create medicine intake schedules based on generated data from PK/PD models.

To provide a comprehensive understanding of our methodology, Fig 1 presents the step-by-step process of our approach from data generation to model optimization.

### Dataset

**Fig 2:**
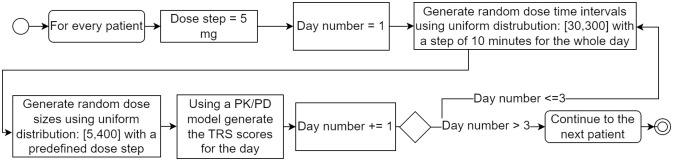
Medication day generation algorithm used to generate 3 days for every patient in the dataset.

The patients’ dataset used in this study was created based on the population characteristics described in [28], which were derived from real PD patients. The reliability of this method in generating patient data has been verified in that study. The population means and covariance matrix were used to generate the characteristics (PK/PD parameters) for 50 PD patients, indicating their individual responses to medication. For every generated patient, data spanning 3 days, including wake-up and falling asleep times was generated using the PK/PD model [27]. The generation algorithm is illustrated in Fig 2.

Using PK/PD models to generate possible courses of patient’s state during the days has been validated in previous publications, demonstrating their ability to accurately model patient’s individual responses to medication. PK/PD models integrate pharmacodynamics and pharmacokinetics to develop mathematical expressions that describe the time course of effect intensity in response to administration of a medicine doses. The PK/PD model used for generating patients’ data consists of 5 equations, 4 of which are differential equations used for calculating the amount of levodopa in 3 compartments—a_0_, a_1_, a_2_ and the concentration in the effect compartment—c_e_ based on patient specific parameters. The final equation is used to calculate the effect (TRS score) based on the concentration in the effect compartment. The TRS values range from -3 (severe symptoms) to 3 (very dyskinetic). These equations are defined as follows and the meanings of the parameters are described in [29].


$$\frac{da_{0}}{dt}=Inf-k_{a}∙a_{0}$$ (1)

$$\frac{da_{1}}{dt}=BIO∙k_{a}∙a_{0}-\left(\frac{Q+CL}{V_{1}}\right)∙a_{1}+\left(\frac{Q}{V_{2}}\right)∙a_{2}+R_{syn}$$ (2)

$$\frac{da_{2}}{dt}=\left(\frac{Q}{V_{1}}\right)∙a_{1}-\left(\frac{Q}{V_{2}}\right)∙a_{2}$$ (3)

$$\frac{dc_{e}}{dt}=kEO∙\left(\frac{a_{1}}{V_{1}}-c_{e}\right)$$ (4)

$$E=BASE+\frac{E_{MAX}\;c_{e}^{\gamma }}{c_{e}^{\gamma }+EC50^{\gamma }}$$ (5)

The parameters of these Eqs (1–5) are also patient specific and have the following meanings:

- Inf—infusion rate (mg/min),
- k_a_—absorption rate (1/min),
- BIO—bioavailability,
- Q—intercompartmental clearance (L/min),
- V_1_, V_2_—volume in first/second compartment (L),
- CL—clearance rate (L/min),
- R_syn_—endogenous levodopa synthesis rate (mg/min),
- kEO—effect rate (1/min),
- BASE—baseline effect—lowest effect value,
- E_MAX_—maximum change from baseline effect,
- EC50—concentration at 50% effect (mg/L),
- γ—Hill coefficient—quantifies how steeply the response changes with increasing medicine concentration.

The BASE and BASE+E_MAX_ are the bounds for the effect values of each patient.

**Fig 3:**
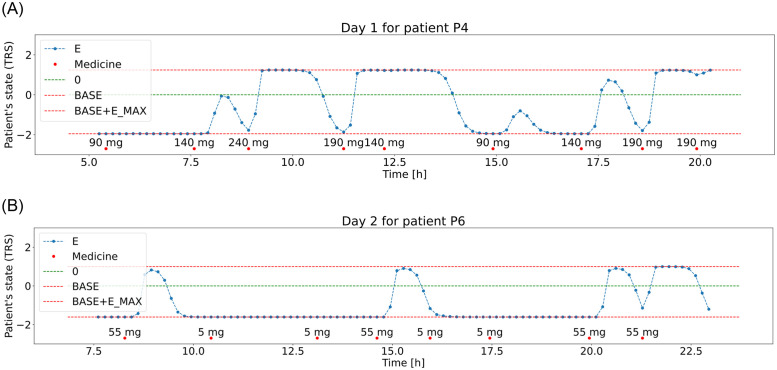
Example medication days generated for patients P4 and P6, medication times (red dots), patient’s state—TRS score (blue line) with bounds for the states (red lines) and optimal state (green line).

The generation process resulted in TRS score values and medication data for 150 days (3 days for each patient) with 10-minute intervals (other time intervals can also be used in the framework), two examples are presented in Fig 3. The dataset was then divided into two sets; 40 patients were used for creating the general medication response model, it would be later fine-tuned to individual patients (remaining 10). The objective of creating the general model is to be able to roughly predict the patient’s future states (TRS score) based on the initial state and taken medication, it will be referred to as the patient state prediction model. This model can be easily retrained to match individual patient needs.

### Patient state prediction model

The model to predict patient’s future states is built using machine learning. For that purpose, two types of using artificial neural networks architecture have been proposed. Both architectures are capable of predicting the course of patient’s states during the day with an equal time step—10 minutes, which was chosen because it provides sufficient precision for dosing medicine (patients usually do not take medicine exactly to the minute) and it is also enough to monitor the changes in the patients TRS score.

The first architecture is a multilayer perceptron [31] referred to as *history model*. A multilayer perceptron is one of the simplest artificial neural networks. It consists of the input layer—first layer receiving the raw input data features, hidden layers—placed between input and output, responsible for transforming input features using weighted computations and activation functions and the output layer—it produces the final prediction of the network. In this problem the network had always one output—to predict the state of the patient. In the multilayer perceptron each neuron takes input from all neurons in previous layers and computes a weighted sum of these inputs.

The created *history model* considers the history of medication for every state prediction. The network is designed to take into account the previous *n* states and the last *k* medication doses to predict the next state to maintain a constant size of input data. The input consists of the *n* values representing previous states and *2k* values representing the times since *k* last doses and *k* sizes of doses. Table 1 presents example network inputs when *n* = 1, *k* = 2 and two medication doses 100 mg taken at 10 minutes and 150 mg taken at 30 minutes.

**Table 1:** Model input for prediction of future patient’s state using two most recent doses and last state of the patient.

Current time	Previous state	Time since last dose	Last dose size	Time since previous dose	Previous dose size
0	s_initial_	0	0	0	0
10	s_0_	0	100	0	0
20	s_10_	10	100	0	0
30	s_20_	0	150	20	100
40	s_30_	10	150	30	100

**Fig 4:**
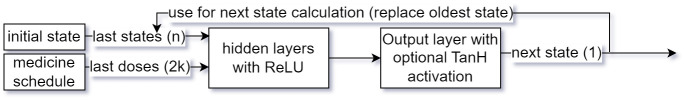
Prediction of states for the whole day provided initial state and medicine schedule using the history model which is based on a multilayer perceptron.

The number of network inputs is equal to n + 2k and there is always one output value representing the next state. In this paper models with n = 1 and k = 2 will be considered. The inference process for the entire day, based on the initial state and medicine schedule is presented in Fig 4. Two variants have been investigated to handle the TRS score range of -3 to 3. In the first variant, there is no activation function in the output layer, the output values are not directly restricted. In the second variant, the output is passed through the hyperbolic tangent (tanh) activation and is then multiplied by the value 3, ensuring that all outputs fall within the range of -3 to 3 (the range for the TRS score). During inference, when *n* > 1 the initial state is replicated to fill all the spots for previous values in the input vector.

The second model is a deep neural network that consists of fully connected and recurrent layers, specifically Long Short-Term Memory (LSTM) layers [32]. Recurrent neural layers are designed to process sequential data and are able to keep track of the history of inputs (previous sequence steps). They introduce loops that allow information to persist over time and data from every step of the sequence is processed using the same set of weights. They are good at capturing temporal dependencies and context; however, they have trouble capturing long-range dependencies. To address this problem LSTMs were introduced. They provide memory cells, with gates that control the flow of information and enable the model to selectively remember or forget information over time allowing it to capture both long and short-range dependencies.

The model created with LSTM cells will be referred to as the *impulse model*. To predict the TRS score in the next step the model requires two values—the previous state and the amount of medicine taken in the previous step. In case no medicine was taken, the value is set to 0. Table 2 presents the input for the impulse model considering the schedule presented in Table 1.

**Table 2:** Model input for prediction of future patient’s state using size of current dose and last state of the patient.

Current time	Previous state	Dose size
0	s_initial_	0
10	s_0_	100
20	s_10_	0
30	s_20_	150
40	s_30_	0

**Fig 5:**
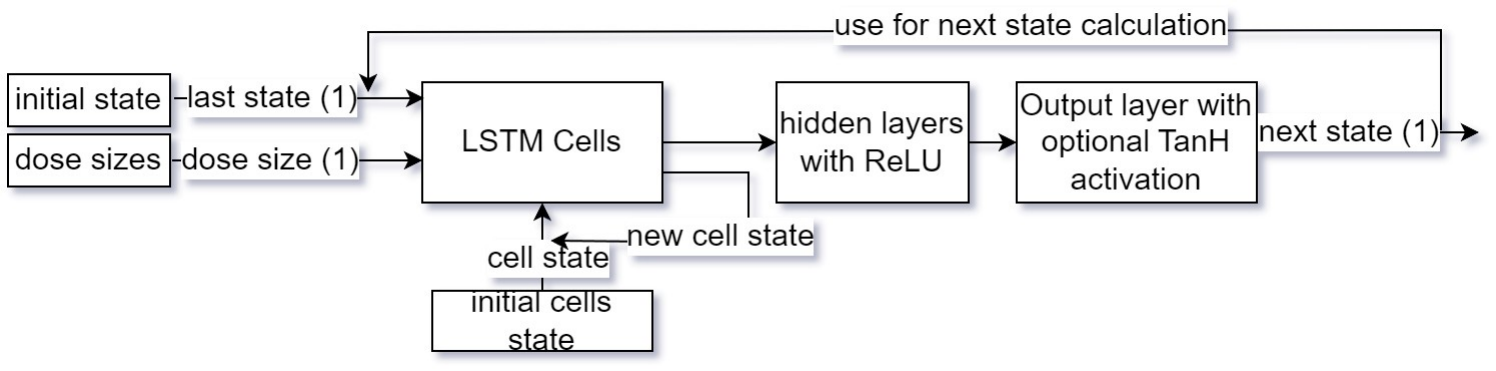
Prediction of states for the whole day provided initial state and medicine doses using the impulse model which uses LSTM cells to keep track of previous states and medicine doses.

The defined impulse model allows capturing the complete history of medication and TRS scores in the LSTM cells’ states without imposing restrictions on the number of considered doses and states. This may lead to improved performance, particularly when multiply doses are taken with small time intervals and the model is expected to be less affected by outliers in the input patient’s states. The process of predicting states for the entire day is presented in Fig 5. The initial LSTM cell state is always 0.

The training process is performed similarly for both models. Each day is treated as a training sample, where the initial (first) state and the medicine schedule serve as input. The remaining states are the expected outputs (for a 16h day, there would be 95 output states). For batch training, all training days are truncated to the same length, which is determined by the shortest day among the patients. The prediction is performed iteratively starting with the first set of inputs (initial state and medication data at t = 0) and each output state is then concatenated with the medication data for subsequent steps. The loss function value is calculated for the entire daily schedule, rather than for each step individually. In both cases the mean squared error was used—to penalize significant deviations from the target values the most. The Adam optimizer [33] is used for both prediction models and the networks are implemented using Keras 2.11.0.

#### General model

In situations where the clinician needs to prescribe accurate and efficient medicine intake schedules, training a model from scratch for each patient can be time-consuming and requires a significant amount of collected data. This paper proposes an alternative approach by creating a general model, which after training is familiar with basic concepts of PK/PD modelling for the PD population. Subsequently, this model is personalized for each individual patient to reflect their specific medication response.

To simulate this behavior the dataset has been split into two parts—patients used to train the general model and remaining patients which will be then used to create personalized models. For fitting the general model, data of 40 patients has been used, each consisting of 3 days. In this section only data of these 40 patients will be considered.

All the 120 days were transformed into the input format for each of the introduced models. The data has been randomly split into training (80%) and validation (20%) sets, resulting in a varying number of days for each of the patients in both sets. To facilitate the training process, the data has then been standardized using the mean and standard deviation of the training set.

Several variations of the defined models were explored to assess their effectiveness. These variants differed in the numbers of neurons in the hidden layers, units in the LSTM layer, the target value—which can be the TRS score or the difference between the next TRS score and the current one and the application of the tanh function at the output of the network. The number of neurons and LSTM cells were chosen as powers of 2, which is a common practice in machine learning and the values were ranging from 16 to 128 for the history model. Lowering the number below 16 resulted in worse performance, while increasing the number beyond 128 did not yield better results and increased the risk of overfitting. The variants of the history model used for training are listed in Table 3 and impulse model in Table 4.

**Table 3:** Variants of history models used for training the general model using data of 40 patients.

Abbreviation	Dense hidden layers	Target value	tanh used
H-16,16	16,16	next state	no
H-16,16, tanh	16,16	next state	yes
H-16,16-diff	16,16	difference	no
H-32,32	32,32	next state	no
H-32,32, tanh	32,32	next state	yes
H-32,32-diff	32,32	difference	no
H-64,64	64,64	next state	no
H-64,64, tanh	64,64	next state	yes
H-64,64-diff	64,64	difference	no
H-128,128	128,128	next state	no
H-128,128, tanh	128,128	next state	yes
H-128,128-diff	128,128	difference	no

**Table 4:** Variants of impulse models used for training the general model using data of 40 patients.

Abbreviation	LSTM units	Dense hidden layers	Target value	tanh used
I-(16)16,16	16	16,16	next state	no
I-(16)16,16, tanh	16	16,16	next state	yes
I-(16)16,16-diff	16	16,16	difference	no
I-(32)32,32	32	32,32	next state	no
I-(32)32,32, tanh	32	32,32	next state	yes
I-(32)32,32-diff	32	32,32	difference	no
I-(8)64,64	8	64,64	next state	no
I-(8)64,64, tanh	8	64,64	next state	yes
I-(8)64,64-diff	8	64,64	difference	no
I-(16)8,8	16	8,8	next state	no
I-(16)8,8, tanh	16	8,8	next state	yes
I-(16)8,8-diff	16	8,8	difference	no
I-(16)32,32	16	32,32	next state	no
I-(16)32,32, tanh	16	32,32	next state	yes
I-(16)32,32-diff	16	32,32	difference	no
I-(32)64,64	32	64,64	next state	no
I-(32)64,64, tanh	32	64,64	next state	yes
I-(32)64,64-diff	32	64,64	difference	no
I-(64)64,64	64	64,64	next state	no
I-(64)64,64, tanh	64	64,64	next state	yes
I-(64)64,64-diff	64	64,64	difference	no

For the training process two callbacks have been defined to perform tasks after every epoch. The first one stops the training process if the value of the loss function on the validation set has not decreased for 10 epochs. The seconds callback is responsible for saving the best model (one with lowest loss on the validation set) achieved during the training process. The networks were trained for 300 epochs with a 0.001 learning rate.

#### Patient specific model

In real-life applications clinicians aim to create accurate medicine schedules with maximum precision while minimizing the amount of data collected from each patient to only what is necessary for the method to be successful. Transfer learning [34] is a machine learning technique that has gained significant attention in recent years due to its ability to improve the performance of a model on a target task by utilizing knowledge learned from a related source task. The basic idea behind transfer learning is to leverage the knowledge and experience gained from solving one problem and apply it to another problem that shows some degree of similarity. Usually, it is performed by first training a network on a similar dataset, that contains more data—for the model to learn the patterns and dependencies in the data. Then the trained network with weights is retrained to adapt more to the target dataset, usually a smaller one, which would not be sufficient to achieve good results if used individually to train. In transfer learning, all of the weights can be updated during retraining (previously set weights are used as a starting point for training) or some of the first layers might be frozen and weights of only a few last layers are updated during the backpropagation of the training process. In case of the history model the whole network was retrained (only two layers), for the impulse model only the fully connected layers were retrained and the weights of the LSTM layers were not updated.

In this case, the previously trained general model is retrained to fit the data for individual patients. The remaining 10 patients are treated as new patients requiring the establishment of a medicine schedule. They are observed for 3 days, during that period the sensor (regarding TRS score) and medication data is collected. The data is then split into training (2 days) and validation (1 day) to perform transfer learning on every model presented in Tables 3 and 4 for each of the 10 patients. The same optimizer and loss functions have been selected (as for training the general model), with a maximum of 100 epochs for training. An early stopping callback has been defined to stop the training if no improved is observed for 25 epochs. Additionally, another callback is set up to save the model every time an improvement is made.

After training, the best results (lowest loss on validation set) are saved. While the training process and the prediction for a single step are not time-consuming tasks, the prediction for multiply steps (entire day) can take long, especially during optimization when it is performed multiply times. To speed up the performance of the prediction for the whole day, the weights from each neural network are extracted and the networks are implemented manually using NumPy array operations, which resulted in a 10–100 times faster (depending on the model) computation of the TRS scores for the day. The significant improvement is attributed to avoiding the overhead of Keras layers for training and inference for big inputs. Using simple array operations can handle the prediction process faster when processing data of smaller size and computations that must be called iteratively and cannot be parallelized (the state result for each step is needed for the computation of the state in the next step).

### Optimization

Designing an optimal medicine schedule is a challenging task, as there is no universally defined objective function in literature, that can accommodate the needs of all patients. During the optimization process, the decision variables represent the times and sizes of all the doses taken throughout the day. In the simplest scenario this would result in *2n* decision variables, with *n* representing the number of doses:

$$T=\left[t_{1},\Delta t_{2},\ldots ,{\Delta t}_{n}\right],\;D=\left[d_{1},d_{2},\ldots .,d_{n}\right],t_{i}\in R_{+},d_{i}\in Z_{+}$$ (6)

*T*—intake times (time of first dose and time intervals between next doses),

*D*—dose sizes.

Instead of defining the T vector as times of consecutive doses, it is defined to include the time of the first dose and the intervals between consecutive doses. This allowed the optimization algorithms to converge faster, approximately 2.3 times faster, due to the changes of individual time variables, which made it easier to define the variable bounds. For the purpose of optimization a framework has been developed that prepares the optimization tasks. Firstly, it allows to enforce some constraints on the decision variables:

1. Forcing the intake times to have discrete values with a defined step e.g., 10 minutes.
2. Making sure that the first dose is taken at wake up.
3. Restricting the optimization to 2 dose sizes—morning dose and equal maintenance doses.
4. Enforcing equal time intervals between doses.

These listed constraints can be combined, leading to a modification in the definition and number of the decision variables. The impact of each constraint, analyzed individually, is presented in Table 5.

**Table 5:** Definition of decision variables, when each of the described constraints is applied. The differences in the dose times and sizes from the original decision variables written in bold.

Constraint number	Dose times	Dose sizes
none	*T* = [*t*_1_,Δ*t*_2_,Δ*t*_3_, Δ*t*_4_], *t*_*i*_ ∈ *R*_+_	*D* = [*d*_1_, *d*_2_, *d*_3_, *d*_4_], *d*_i_ ∈ *Z*_+_
1	*T* = [*t*_1_,Δ*t*_2_,Δ*t*_3_, Δ*t*_4_], *t*_*i*_ ∈ ***Z***_**+**_	*D* = [*d*_1_, *d*_2_, *d*_3_, *d*_4_], *d*_i_ ∈ *Z*_+_
2	*T* = [Δ***t***_2_,Δ***t***_3_, Δ***t***_4_**]**, *t*_*i*_ ∈ *R*_+_	*D* = [*d*_1_, *d*_2_, *d*_3_, *d*_4_], *d*_i_ ∈ *Z*_+_
3	*T* = [*t*_1_,Δ*t*_2_,Δ*t*_3_, Δ*t*_4_], *t*_*i*_ ∈ *R*_+_	*D* = [***d***_***mor***_, ***d***_***main***_], *d*_*i*_ ∈ *Z*_+_
4	*T* = [*t*_1_, **Δ*t***], *t*_*i*_ ∈ *R*_+_	*D* = [*d*_1_, *d*_2_, *d*_3_, *d*_4_], *d*_i_ ∈ *Z*_+_

Algorithms that will be used for optimization will also require defining the bounds for each variable, following previous publications [29], the smallest time interval is set to 90 minutes, and the maximum value is defined by the number of doses and the day length for the patient. The dose sizes can vary between 0 and 400 mg.

To optimize the medicine schedule the framework establishes the following criteria to define the objective functions:

- Minimizing the sum of doses
$$\text{min}\;\sum_{i=1}^{n}d_{i}$$ (7)
- Minimizing the sum of squares of difference between the states and optimal state
$$\text{m}\text{in}\;\int_{t_{u}}^{t_{d}}{\left(st\left(t,T,D\right)-\theta \right)}^{2}dt$$ (8)
- Minimizing the area outside the target range
$$\text{m}\text{in}\;\int_{t_{u}}^{t_{d}}{\text{m}\text{ax}}^{2}\left(0,\theta_{min}-st\left(t,T,D\right),st\left(t,T,D\right)-\theta_{max}\right)dt$$ (9)
- Minimizing the area below the threshold
$$\text{m}\text{in}\;\int_{t_{u}}^{t_{d}}{\text{m}\text{ax}}^{2}\left(0,\theta_{t}-st\left(t,T,D\right)\right)dt$$ (10)

*n*—number of doses,

*d*_*i*_—size of dose *i*,

θ—optimal patient state,

θ_*min*,_ θ_*max*_—the lower and upper bound of the target patient range,

θ_*t*_—the threshold patient state,

*st(t*, *T*, *D)*—patient state function, represents the state of the patient at time *t* under the schedule represented by *T* (intake times) and *D* (dose sizes).

The criteria can be used individually or combined with custom weights what, providing greater flexibility during the optimization process and potentially leading to better results.

Given the non-linear character of the objective functions (8–10) (the value is calculated using an artificial neural network or PK/PD model and the functions have a quadratic nature) only a subset of optimization algorithms is suitable. In this case, heuristic algorithms from the evolutionary algorithms class have been selected. The genetic algorithm [35] and differential evolution [36] have been chosen as they provided satisfactory results in solving this problem. These algorithms can handle non-linear objective functions and constraints, they also do not fall into local minimums, due to the use of mutation operators. Used algorithms iteratively try to improve candidate solutions to improve the value of the objective function. It is performed using evolutionary algorithm operators that are inspired by natural selection.

The genetic algorithm was implemented in Python using the DEAP library [37]. It offers convenient utilities to perform the optimization and includes operators for selection, crossover and mutation. The library is easily extensible and provides a simple interface for modifying or creating custom operators. It also allows for constructing individuals consisting of variables of different types, which is necessary to handle discrete dose sizes and continuous intake times.

The framework that has been created has an interface supporting DEAP and directly provides the fitness function (in this case it is defined as the negative value of the objective function) and the definition of the decision variables—their datatype and bounds, that indicate the minimum and maximum values for each variable. As explained before the datatype and meaning of the decision variables is dependent on the selected constraints from Table 5. The number of decision variables can be *2n* (where *n* represents the number of doses)—when no additional constraints are imposed. However, when all constraints are applied, it can be reduced to just 3—[Δ*t*, *d*_*mor*_, *d*_*main*_]. Adding these constraints makes the dosing process easier and simpler to follow, but the created schedule will not be as flexible and the patient might experience more or PD symptoms and levodopa induced dyskinesia during the treatment. When determining the dosing schedule, a compromise must be made between the usability of the suggested therapy and the patient’s condition throughout the day.

To create a genetic algorithm individual the decision variables arrays—intake times and dose sizes are concatenated. This means that the times will always be placed in the beginning of the array representing the individual and the dose size information will be in the back e.g. [*t*_1_,Δ*t*_2_,*d*_1_,*d*_2_], [Δ*t*, *d*_*mor*_, *d*_*main*_]. The composition of the individual (dose schedule) varies based on the number of constraints applied. It can consist solely of discrete variables (when constraint 1 is applied) or a combination of continuous variables (first part of the array) and discrete variables (second part of the array). This distinction should be considered when selecting and applying the operators. The optimization task utilizes the following operators:

- Crossover—Two-point crossover—the algorithm randomly chooses two indexes in the individual that indicate where two individuals should be crossed over producing two new individuals.
- Mutation—applied to each variable in the individual with a predefined probability:
  - Gaussian mutation for continuous variables (intake times)—the value of the variable is updated with the value generated with the Gaussian distribution (specified mean and standard deviation). To ensure that the value remains within predefined bounds, a check is performed after each mutation. If the condition is not satisfied the value is adjusted to the exceeded bound.
  - Integer uniform mutation for discrete variables—the value of the decision variable is replaced with a new integer value generated from a uniform distribution within the specified bounds.
- Selection—Tournament selection—randomly picks *k* individuals and selects the best individual based on the value of the fitness function.

**Fig 6:**
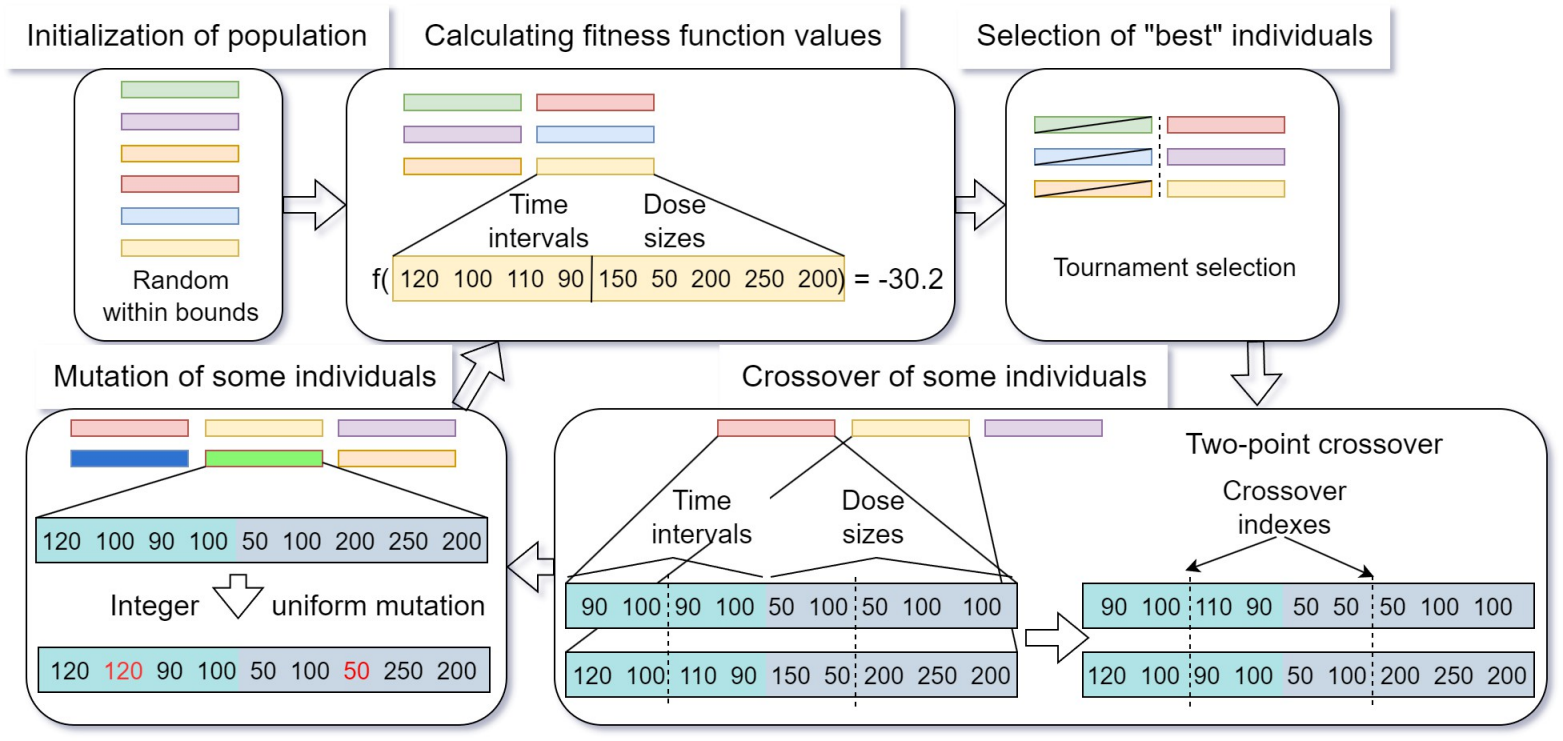
Steps (application of operators) in the genetic algorithm used to optimize the medicine intake schedules.

The implemented genetic algorithm utilizes operators outlined in Fig 6.

**Fig 7:**
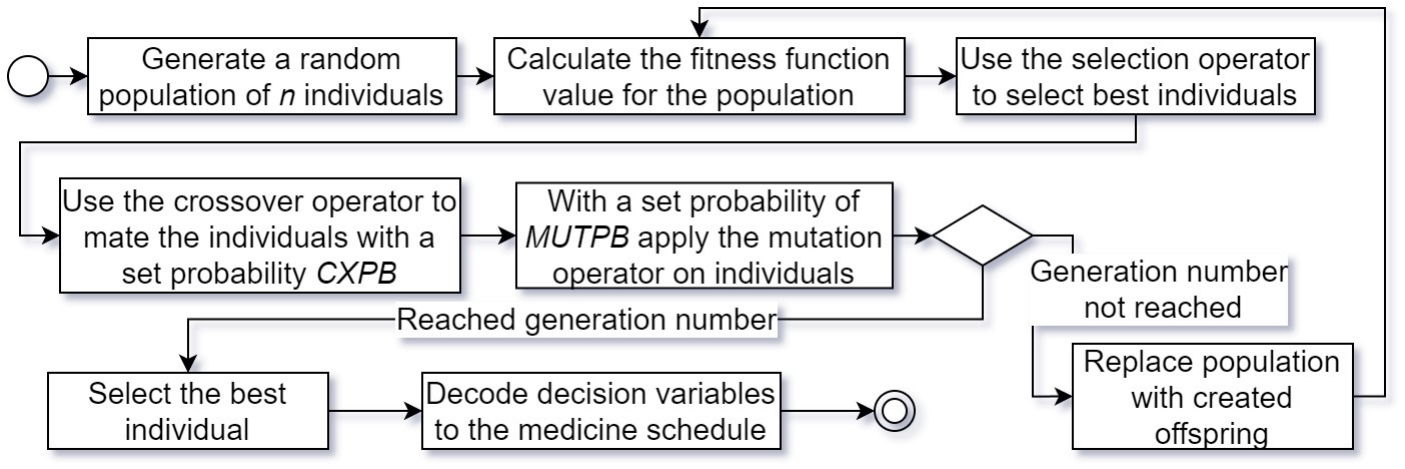
Execution of the genetic algorithm for medicine schedule optimization in PD.

The crossover operation is performed with a probability of 0.5, while the mutation operation has a probability of 0.2. Each variable undergoes mutation with probability of 0.1. The selected operators are simple, fast to apply and allow the algorithm to quickly find optimal and suboptimal solutions, thus outperforming more complex operators. The overall execution of the algorithm is presented in Fig 7.

The size of the initial population and the number of generations for optimizing the medicine intake schedules depended on the number of decision variables. However, the population size never exceeded 200 and the number of generations was limited to 100.

**Fig 8:**
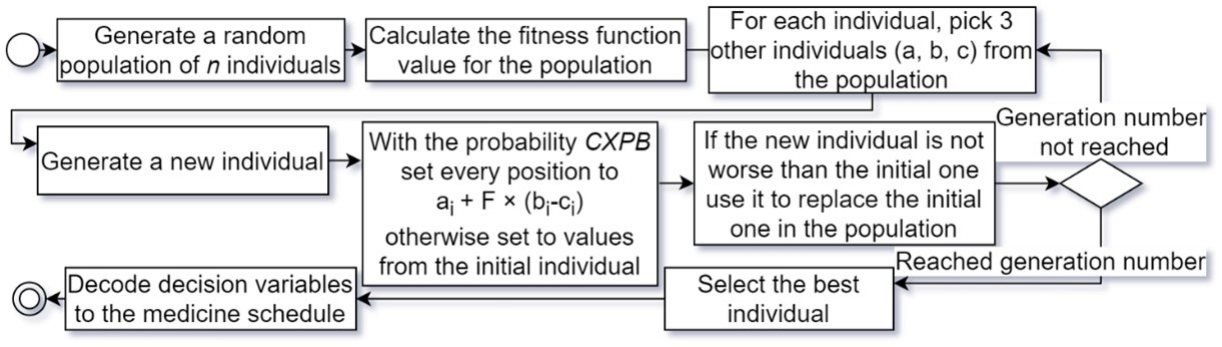
Execution of the differential evolution algorithm for medicine schedule optimization in PD.

Differential evolution, which is also an example of evolutionary algorithms, is a powerful optimization algorithm that has gained widespread popularity due to its simplicity, efficiency, and effectiveness in solving complex optimization problems. The algorithm is based on the principle of mutation and selection, where candidate solutions are perturbed by a set of differential vectors to generate a trial solution. The trial solution is then compared with the current best solution, and the fitter of the two is selected as the parent for the next generation. The algorithm requires the specification of 3 parameters: the population size (n), crossover probability (CXPB) and the differential weight (F). The steps of the algorithm are outlined in Fig 8.

The medicine schedule optimization utilized the *differential_evolution* implementation from Python’s *SciPy 1*.*10*.*1* library. It provides a simple interface, requiring the objective function callback and the decision variable bounds and also accepts other parameters including the *integrality* constraint, determining which of the decision variables are constrained to integers. By combining the *integrality* and *bounds* parameters the constraints from Table 5 were applied. The parameters to the algorithm were supplied by the previously designed framework, which just like in the case of genetic algorithm takes care of coding and decoding decision variables. The pseudocode for both, the genetic algorithm (left) and differential evolution (right), has been presented below.

**Table:** 

**Input**: n > 2, g_max_ **Initialize:** the population *x* with *n* vectors sampled at random; **Set**: g = 0 **While** g ≤ g_max_ **Do** x = select(x) **For** i = 1 **to** n/2 **Do** **If** rand(0, 1) < CXPB **Then** crossover(x[2i], x[2i+1]) **End If** **End For** **For** i = 1 **to** n **Do** **If** rand(0, 1) < MUTPB **Then** mutate(x[i]) **End If** **End For** g = g + 1 **End While**	**Input**: F > 0, n > 2, g_max_, m > 1 f—objective function **Initialize:** the population *x* with *n* vectors of length *m* sampled at random; **Set**: g = 0 **While** g ≤ g_max_ **Do** **For** j = 1 **to** n **Do** Select distinct individuals *a*, *b*, *c* from *x* at random, *a* ≠ *b* ≠ *c* ≠ *x[j]* **For** i = 1 **to** m **Do** v[i]=a[i]+F*b[i]-c[i]ifrand(0,1)<CXPBx[j,i]otherwise **End For** **If** f(v) ≤ f(x[j]) **Then** x[j] = v **End If** **End For** g = g + 1 **End While**

Various experiments were conducted with other algorithms and combinations of them; however, they did not provide significantly better results or performance and the libraries providing these algorithms were less clear and documented. Both algorithms were used for schedule optimization; however, only the best result is showcased in the paper.

### Reinforcement learning

Reinforcement learning (RL) [38] is a subfield of machine learning that deals with how an agent should learn to make decisions through trial and error. In our system, RL is employed to adaptively learn the most efficient medication schedules for patients based on real-time data and predictive models.

In reinforcement learning, an agent interacts with an environment and learns by receiving feedback in the form of rewards or penalties for its actions. The agent’s goal is to maximize its cumulative reward over time, which is often referred to as the “return.”

The agent in our RL model is trained to make decisions on the optimal time and dose size of medication based on patient-specific parameters. RL algorithms typically use a policy, which maps states to actions, to make decisions. The policy is learned through exploration of the environment and exploitation of past experiences. The agent’s actions in the environment are guided by the policy, which is updated based on the feedback received from the environment.

The application of RL in creating individualized medicine intake schedules has been previously presented in a publication [30]. This paper applies RL to learn the optimal strategies to take medications—time and size of doses. One of the main challenges in using RL for this task is correctly defining the environment. In this case, the environment is constructed based on two types of models: PK/PD model, ML model, both created to predict the patient’s state in future under a specific medication schedule. In case of optimization the course of the TRS scores for the whole day was created at once, for RL, the models have a wrapper which allows generating values step by step (step size equal to 10 minutes). The environment was created using the *Gymnasium* library, a fork of the OpenAI’s Gym library [39].

The library required the definition of reset() and step() methods, which are called to reset the state of the environment and make a next step by taking an action, as well as the observation and action space. The action space was defined as single integers from 0 to *n*_*max*_ representing dose sizes of medicine. The number of available actions is patient specific and depends on the dose step, e.g., a patient with maximum dose of 400 mg and a dose step of 25 mg would have 17 available actions: 0—no medication, 1–25 mg, 2–50 mg, …, 16–400 mg. The shape and interpretation of the observation space varied depending on the model that was used. In the PK/PD model, observations were represented by the amount/concentration of medicine in different compartments a_0_, a_1_, a_2_, c_e_, resulting in a 4-variable environment state. For the history model, the state of the environment consisted of *2k+1* variables, where *k* represents the number of recent doses considered. The variables captured the resent patient’s state, the sizes of *k* previous doses and the elapsed time since their administration. For the impulse model, the LSTM cells capture and process temporal patterns in the patient’s condition. The LSTM’s internal states serve as the observation space for the RL model. This allows the RL agent to base its decisions on a richer, time-aware representation of patient conditions. Due to the use of tanh function in LSTM cells, the bounds for the variables were set -1 to 1.

The reset method is expected to reset the state of the environment and return the initial observation and the auxiliary info dictionary which helps in result interpretation. In the presented implementation, the environment collects the information about past patient’s states and administered medication doses since last reset. These values, along with previously defined environment state and time are reset upon invoking the reset method.

The step method takes an action as an argument, which is then translated into the appropriate dose and then the input for the state prediction model is prepared. The TRS score for the next step is calculated and if it exceeds a specified maximum value for the patient the episode is terminated—the value is patient specific. For every patient a target TRS score range has been selected and the reward is the negative value of the objective function (9). The episode is terminated when the patient’s state exceeds the maximum value or after reaching 110 steps, equivalent to a duration of over 18 hours (typically longer than the time patient is awake. The method returns the current observation, the reward for the step, boolean values indicating whether the episode was terminated or truncated and additional information.

Depending on the selected dose step, the RL agent may generate medicine intake schedules that expect the user to take small medicine doses at each time step (e.g., every 10 minutes). To make the model’s suggestions more applicable in real-life situations, we have implemented a constraint that imposes a minimum time interval between medication doses. After a dose is taken, the agent must wait a predefined number of time steps before taking the next action. In the environment implementation, this is handled by a loop simulating several steps in the step() function when a dose is taken (assuming the agent selects an action other than 0).

**Fig 9:**
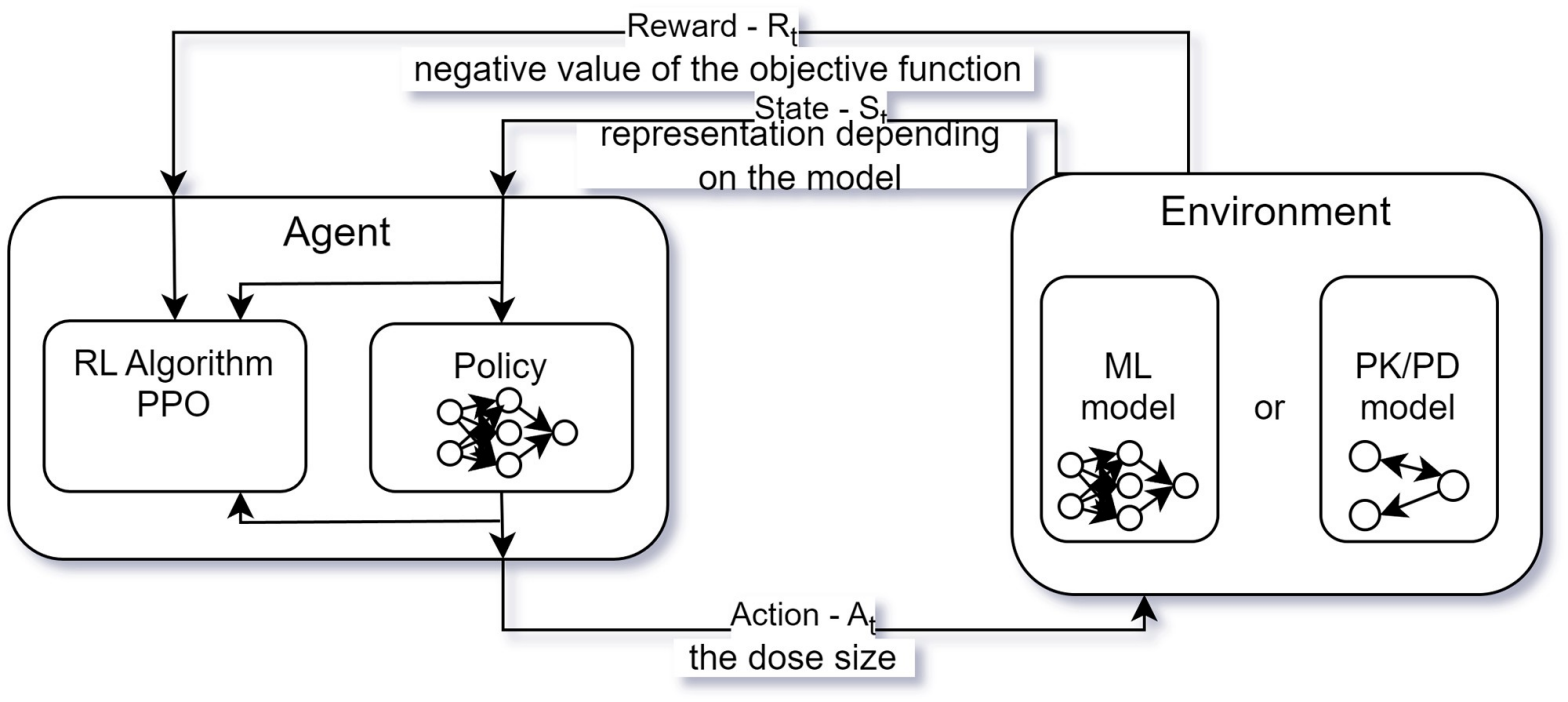
The process of training the RL agent to suggest appropriate medicine doses in different patient’s conditions using the PK/PD or ML model as the environment.

The network training process utilized the Stable Baselines3 library [40]. It offers implementations of multiply reinforcement learning algorithms, including both on-policy and off-policy approaches. The applications of algorithms differ and depend on the definition of the action space. When handling discrete action spaces, the following algorithms can be applied: A2C (on-policy), DQN (off-policy) and PPO (on-policy). After conducting trials, it was determined that PPO provided the best results. Therefore it was the only algorithm chosen to create schedules for all the patients. The process of training the RL agent is presented in Fig 9.

## Results

This chapter presents achieved results for the patient state prediction models, both the general and the patient specific model and the results achieved finding best medicine schedules using optimization and RL. These results are then compared with the ones acquired with the method described in [29].

### Patient state prediction model

#### General model

To select the best model for predicting the patient’s medicine response all the models from Tables 3 and 4 were trained ten times—to minimize the influence of the model’s initial weights. The chosen number of epochs—300 was sufficient for training. In most cases the training process was stopped with the early stop callback, since there was no improvement during last epochs. Despite training the general model on data from many patients, the process of training was fast and resulted in well-fitted models, considering the diversity of patients. To evaluate and compare the performance of the trained models, the following metrics were calculated:

- Mean squared error (MSE), which also serves as the loss function (smaller value is preferred):
$$MSE\left(y,\hat{y}\right)=\frac{1}{n}\sum_{i=1}^{n}{\left(y_{i}-\hat{y_{i}}\right)}^{2}$$ (11)
- Mean absolute error (MAE) (smaller value is preferred):
$$MAE\left(y,\hat{y}\right)=\frac{1}{n}\sum_{i=1}^{n}{\left|y_{i}-\hat{y_{i}}\right|}^{2}$$ (12)
- The coefficient of determination (R^2^) [41] (greater value is preferred):
$$R^{2}\left(y,\hat{y}\right)=1-\frac{\sum_{i=1}^{n}{\left(y_{i}-\hat{y_{i}}\right)}^{2}}{\sum_{i=1}^{n}{\left(y_{i}-\bar{y_{i}}\right)}^{2}}$$ (13)
  Where *y*_*i*_—true value, $\hat{y_{i}}$—predicted value, $\bar{y_{i}}$—mean of true values.

Table 6 presents the results for the history models, displaying the mean values across the 10 training rounds after removing any outliers for both the training and validation sets. When evaluating the model, it is important to observe low loss function values on both the validation and the training set. The models should not be prone to overfitting (retraining on a small dataset for individual patients may lead to it in case of complex network structures).

**Table 6:** Mean metrics values—Mean squared error (MSE), mean absolute error (MAE) and coefficient of determination (R^2^) for history models sorted by MSE in validation set. Best results for each metric presented in bold.

Model info	Training set			Validation set		
	MSE	MAE	R^2^	MSE	MAE	R^2^
H-128,128-diff	0.254	0.373	0.683	**0.310**	0.406	0.621
H-32,32-tanh	0.281	0.384	0.666	0.315	0.401	**0.625**
H-64,64-tanh	0.284	0.385	0.660	0.315	0.404	0.624
H-64,64-diff	0.279	0.382	0.668	0.322	0.410	0.620
H-64,64	**0.230**	**0.345**	**0.728**	0.324	**0.395**	0.621
H-128,128-tanh	0.282	0.384	0.661	0.325	0.403	0.614
H-128,128	0.242	0.355	0.713	0.325	0.396	0.618
H-16,16-tanh	0.296	0.398	0.641	0.326	0.410	0.609
H-16,16-diff	0.306	0.398	0.645	0.327	0.408	0.623
H-32,32-diff	0.266	0.370	0.683	0.330	0.401	0.607
H-16,16	0.313	0.406	0.635	0.332	0.415	0.610
H-32,32	0.272	0.388	0.679	0.333	0.417	0.609

To further assess the performance of the models, the Wilcoxon signed-rank test [42], a non-parametric statistical test, was employed. This test examines the null hypothesis that two related paired samples are drawn from the same distribution. It provides the option to choose from three alternative hypothesis:

- Greater—can determine if one set of measurements is stochastically greater than the other set of measurements,
- Less—helps determine if one set of measurements is stochastically less than the other set of measurements,
- Two-Sided—checks whether there is a significant difference between the two distributions.

**Fig 10:**
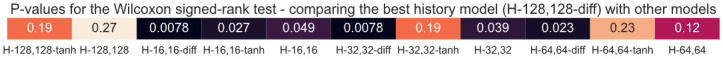
Results of p-values from the Wilcoxon signed-rank test which was performed to verify which models are significantly worse than the best history model. The test was conducted with the alternative hypothesis that the distribution underlying one set of measurements (mean squared errors of the best model) is stochastically greater than the distribution underlying the second set of measurements (mean squared errors of other models).

In this analysis, the test was conducted using the greater alternative hypothesis. The best performing model was compared with the remaining to determine if it was significantly better—had significantly lower MSE. The resulting p-values from the Wilcoxon signed-rank test, comparing the MSE values in 10 trials of the best model with the remaining models, are illustrated in Fig 10. With a significance level of 0.05, it was observed that the best model (H-128,128-diff), significantly outperformed 6 models, p-values lower than the significance level resulted in rejecting the null hypothesis, thus accepting that the MSEs of these models are significantly greater than the MSEs of the best model (alternative hypothesis).

**Fig 11:**
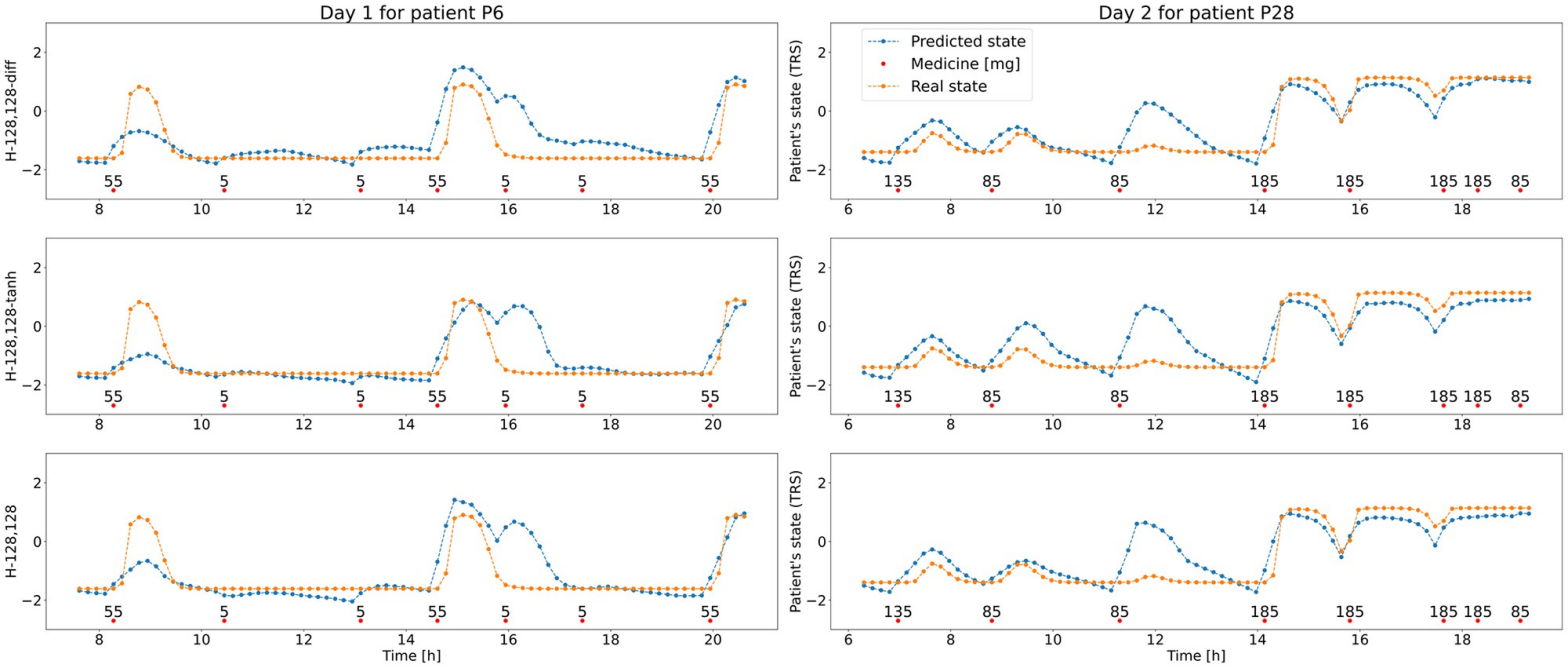
Day 1 for patient P6 and day 2 for patient P28 with medicine doses (red dots) and changing TRS score values during day, the “real”—generated with PK/PD model (orange) and predicted(blue) with top 3 history ML models—H-128,128-diff (top), H-128,128-tanh (middle) and H-128,128 (bottom).

For further use, to reduce the training time and focus on getting best results in fitting to individual patients only 5 history models with the lowest MSE were selected. What is noticeable, is that the models predicting the difference between the next patient’s state and the current one perform slightly better than the models that predict the next value of the state. The restriction of the outcome values using the tanh function also seems to improve the performance of the network. The number of neurons in the hidden layers does not seem to have an explainable influence on the performance of the history networks. Two patient days have been selected from the validation set to visually compare the outcomes of the top 3 models (with lowest validation MSE) and they are presented in Fig 11.

Difficulties with reflecting the individual patient’s response to the medication occurred in general models. When the patient is more medicine-resistant and small doses are taken, there should be no drastic change in the patient’s state. However, these models seem to react to even small doses such as 5 mg. This proves the need for individualization of medicine schedules, since a dose of some size might have a small impact on one patient and a significant one on the other. Due to the restricted length of the history in the history model, only two last doses and two last states are considered when calculating the next state. The model demonstrates suboptimal performance in situations characterized by frequent doses, small time intervals and significant fluctuations in the patient’s recent states.

For the impulse model a different set of architectures has been chosen. In this case also 10 training cycles have been performed for each of the models (to reduce the influence of randomly initialized weights) and their results—the means of MSE, MAE and R^2^ have been presented in Table 7.

**Table 7:** Mean metrics values—Mean squared error (MSE), mean absolute error (MAE) and coefficient of determination (R^2^) for impulse models sorted by MSE in validation set. Best results for each metric presented in bold.

Model info	Training set			Validation set		
	MSE	MAE	R^2^	MSE	MAE	R^2^
I-(32)32,32-tanh	0.283	0.378	0.671	**0.255**	0.355	**0.686**
I-(8)64,64-diff	0.258	0.368	0.698	0.257	0.358	0.680
I-(16)16,16	0.240	0.353	0.708	0.258	**0.349**	0.680
I-(32)32,32-diff	0.214	0.326	0.742	0.260	0.360	0.676
I-(8)64,64	0.260	0.372	0.685	0.261	0.360	0.660
I-(32)32,32	0.242	0.348	0.704	0.262	0.364	0.663
I-(16)32,32-tanh	0.249	0.357	0.711	0.264	0.361	0.672
I-(32)64,64-tanh	0.251	0.359	0.704	0.268	0.370	0.666
I-(32)64,64-diff	0.198	0.321	0.765	0.270	0.353	0.677
I-(64)64,64-diff	0.219	0.334	0.736	0.271	0.369	0.666
I-(16)8,8	0.282	0.384	0.665	0.273	0.374	0.653
I-(16)16,16-diff	0.213	0.335	0.739	0.274	0.351	0.664
I-(16)8,8-tanh	0.242	0.359	0.706	0.274	0.353	0.671
I-(16)32,32-diff	**0.171**	**0.299**	**0.793**	0.274	0.361	0.664
I-(64)64,64	0.196	0.316	0.761	0.275	0.376	0.658
I-(16)32,32	0.282	0.390	0.670	0.275	0.383	0.660
I-(64)64,64-tanh	0.260	0.366	0.689	0.275	0.374	0.652
I-(32)64,64	0.280	0.382	0.661	0.276	0.382	0.649
I-(8)64,64-tanh	0.255	0.366	0.694	0.277	0.371	0.637
I-(16)16,16-tanh	0.254	0.361	0.702	0.278	0.372	0.658
I-(16)8,8-diff	0.251	0.368	0.663	0.292	0.374	0.572

**Fig 12:**
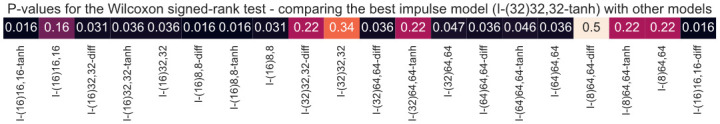
Resulting p-values from the Wilcoxon signed-rank test performed to verify the models that are significantly worse than the best impulse model. The test was conducted with the alternative hypothesis that the distribution underlying one set of measurements (mean squared errors of the best model) is stochastically greater than the distribution underlying the second set of measurements (mean squared errors of other models).

The Wilcoxon signed-rank was also applied to compare the performance of impulse models and evaluate the significance of the observed differences in MSE. The resulting p-values from comparing the best model (I-(32)32,32-tanh) with other models are presented in Fig 12. Using a significance level of 0.05, it was found that the model significantly outperforms 13 models.

The impulse models, due to the use of LSTM cells, were able to capture the whole available history, all the provided previous states, previously administered doses. This resulted in a significantly better performance on the validation set, the worst performing model from Table 7 has lower MSE and MAE than the best model from Table 6. This proves the advantage of using LSTMs for analyzing time series. However, this approach allows handling only doses that were taken at time steps—times of doses taken between them would have to be rounded to match a value provided by the time step (e.g., dose taken at 8:17 would be treated as taken at 8:20). This might lead to some inconsistencies in training the model on real samples, when the patient might take the medicine at a different time. To avoid this lower time steps could be chosen, or another feature could be added to the vector representing minutes since the dose was taken.

**Fig 13:**
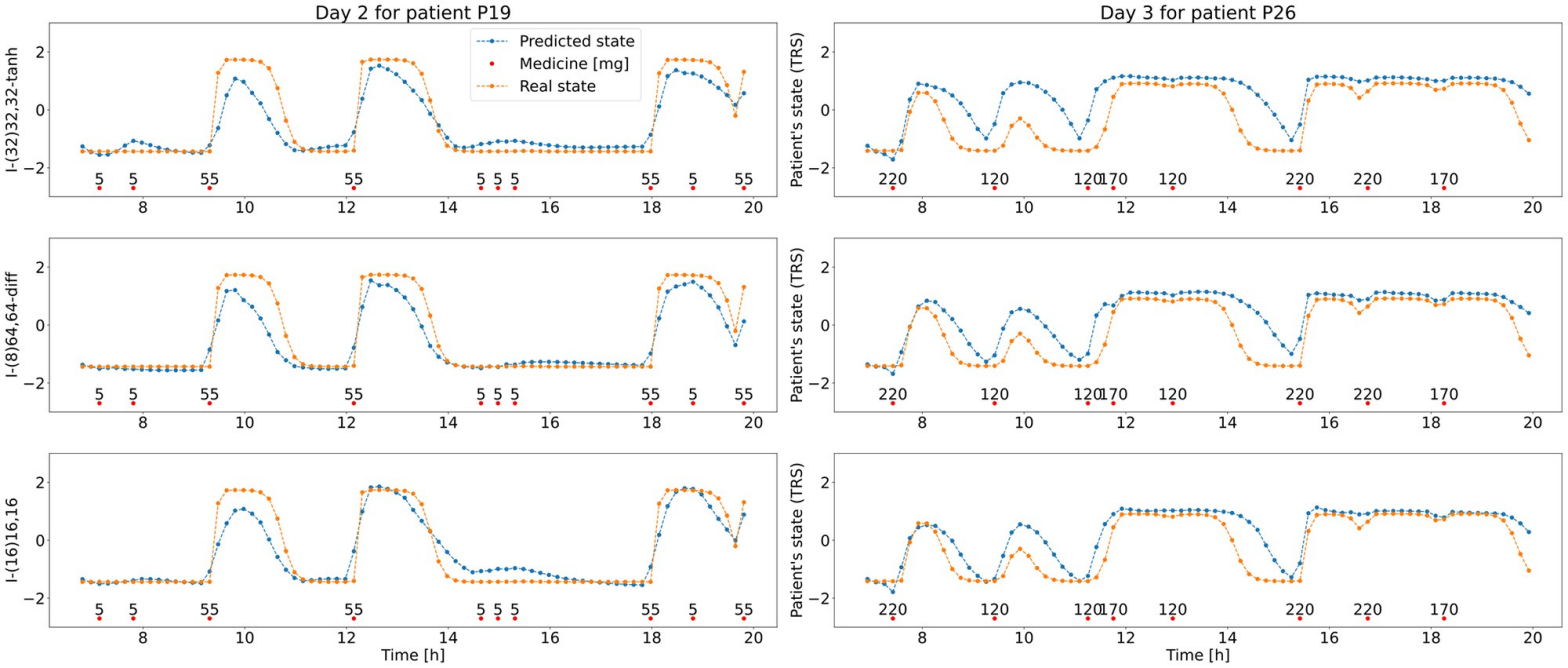
Day 2 for patient P19 and day 3 for patient P26 with medicine doses (red dots) and changing TRS score values during day, the “real”—generated with PK/PD model (orange) and predicted(blue) with top 3 impulse ML models—I-(32)32,32-tanh (top), I-(8)64,64-diff (middle) and I-(16)16,16 (bottom).

Using the best 3 models, days for two patients from the validation set have been created. The results for each of the networks do not differ as much as in the case of history models and they match the target values better. The results are presented in Fig 13.

#### Patient specific model

To retrain and create patient specific models, only top 5 history (Table 6) and top 5 (Table 7) impulse models were used. For each of the 10 remaining patients, each of these models was trained with 2 generated days and the third day was treated as the validation set. Table 8 presents the results of the training process providing the metrics for the general model (before retraining) and individualized model (after retraining) for best performing models (lowest MSE for training and validation sets) for every patient.

**Table 8:** Metrics values—Mean squared error (MSE), mean absolute error (MAE) and coefficient of determination (R2) for individual patients’ training (day 1 and 2) and validation data (day 3) before and after retraining.

P	Best model	Before retraining—training (validation)			After retraining—training (validation)		
		MSE	MAE	R^2^	MSE	MAE	R^2^
41	I-(32)32,32-diff	0.136 (0.296)	0.296 (0.390)	0.688 (0.539)	0.000314 (0.00683)	0.0124 (0.0467)	0.999 (0.989)
42	I-(8)64,64	0.0969 (0.500)	0.186 (0.513)	0.870 (0.716)	0.00262 (0.0274)	0.0264 (0.0841)	0.996 (0.984)
43	I-(32)32,32-tanh	0.244 (0.188)	0.321 (0.270)	0.734 (0.786)	0.0000342 (0.0100)	0.00454 (0.0586)	0.999 (0.989)
44	I-(16)16,16	0.452 (0.313)	0.485 (0.351)	-1.56 (0.255)	0.00144 (0.00763)	0.0200 (0.0469)	0.992 (0.982)
45	I-(32)32,32-tanh	0.331 (0.0911)	0.447 (0.270)	0.518 (0.828)	0.00235 (0.00982)	0.0314 (0.0679)	0.997 (0.981)
46	I-(8)64,64	0.145 (0.126)	0.323 (0.298)	0.774 (0.846)	0.00324 (0.0110)	0.0388 (0.0596)	0.995 (0.987)
47	I-(16)16,16	1.55 (0.157)	0.837 (0.300)	-0.866 (0.821)	0.000639 (0.00412)	0.0193 (0.0372)	0.999 (0.995)
48	I-(8)64,64	0.441 (0.505)	0.540 (0.543)	0.590 (0.648)	0.000855 (0.00853)	0.0227 (0.0529)	0.999 (0.994)
49	I-(8)64,64	0.397 (0.622)	0.405 (0.548)	0.456 (-0.0342)	0.000302 (0.0214)	0.0132 (0.0735)	0.999 (0.964)
50	I-(8)64,64	0.961 (1.07)	0.706 (0.870)	0.174 (-2.31)	0.00128 (0.00579)	0.0278 (0.0483)	0.999 (0.982)

**Fig 14:**
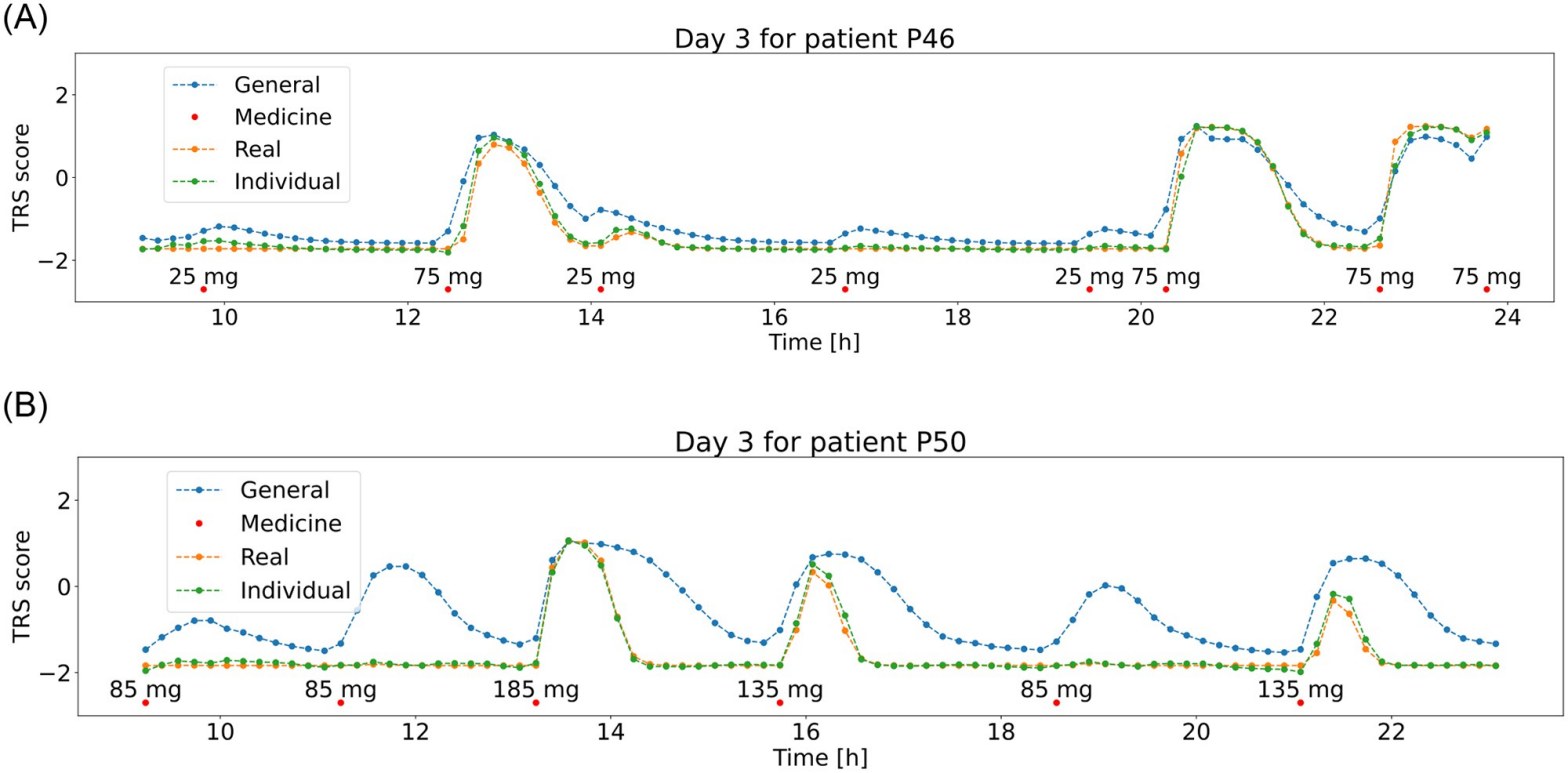
Day 3 for patients P46 (top) and P50 (bottom) with medicine doses (red dots) and changing TRS score values during day—the “real”—generated with PK/PD model (orange), predicted before retraining(blue) and after retraining(green) with the top ML model.

The metric values for the general model for patients that have never been seen are not satisfying. The errors are significantly higher than for the validation set in the previous training and in some cases the R^2^ has even negative values, which concludes that this model does not reflect the medication response for specific patients well. After retraining the models on two days for every patient, the results have greatly improved with the worst results for patient 49, with MSE = 0.0214 and R^2^ = 0.964 and they are expected to be effective in predicting the patient’s response to medication. Fig 14 presents the best model’s performance for validation days for patients 46 and 50 showing how the model’s output has improved after retraining.

Table 8 shows that different models have been selected as best for each of the patients and a method was selected for choosing the best one overall—the mean metrics among patients have been calculated and the model with lowest mean validation MSE was considered the best, mean MSE and other metrics for these models are presented in Table 9.

**Table 9:** Mean metrics values—Mean squared error (MSE), mean absolute error (MAE) and coefficient of determination (R^2^) for individual models sorted by validation MSE. Best results for each metric presented in bold.

Model info	Training set			Validation set		
	MSE	MAE	R^2^	MSE	MAE	R^2^
I-(8)64,64	0.00756	0.0423	0.984	**0.0215**	**0.0795**	0.965
I-(8)64,64-diff	0.0076	0.05	0.989	0.0239	0.0933	**0.967**
I-(16)16,16	0.0154	0.0716	0.979	0.0438	0.115	0.957
I-(32)32,32-tanh	0.00621	0.0427	0.983	0.0466	0.116	0.918
I-(32)32,32-diff	**0.00434**	**0.0359**	**0.995**	0.05	0.123	0.906
H-64,64-tanh	0.0226	0.0921	0.965	0.0853	0.193	0.858
H-32,32-tanh	0.0524	0.141	0.897	0.0915	0.208	0.854
H-64,64	0.0368	0.122	0.935	0.0967	0.21	0.832
H-128,128-diff	0.0727	0.163	0.826	0.107	0.226	0.804
H-64,64-diff	0.119	0.237	0.834	0.133	0.263	0.809

Presented results show that the impulse model with 8 units in the LSTM cell and 2 hidden fully connected layers provided the best results on the validation dataset for each of the patients, this suggests that this model might be used for new patients. The table also shows the advantage of impulse models compared to history models which had the lowest results for all the metrics. Considering the results presented in this table, model I-(8)64,64 was chosen for optimization, since it provided the best performance among the patients.

**Fig 15:**
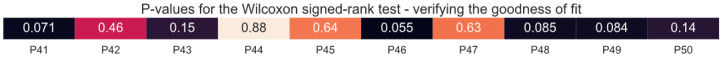
Resulting p-values from the Wilcoxon signed-rank test performed to verify that the individual models are well-fitted (same distributions of target and predicted values). The test was conducted with the two-sided alternative hypothesis.

To verify the goodness of fit of the selected best models, the Wilcoxon signed-rank test was used to compare the target values (generated from the PK/PD model) with the ML model predictions on the validation set. This test is recommended in the performance validation of simulation models [42]. The null hypothesis for the test assumes that two related paired samples (target and predicted values) come from the same distribution. The resulting p-values for the test are presented in Fig 15. Across all patients, the null hypothesis was accepted at a significance level of 0.05, confirming that the models are well-fitted.

### Generating possible schedules

In order to compare results obtained from optimization and reinforcement learning with previously published results, the algorithm introduced in [29] was used to find medicine intakes schedules for each of the patients. This required the specification if 2 parameters for each patient:

- Target range—range defining the minimum and maximum target state, defining the desired patient’s state.
- Threshold—the minimum patient’s state that should not be exceeded before the next dose is taken.

The find the best schedule for the patient, the objective was to minimize the area outside the target range (9) while ensuring the patient’s state did not fall below the threshold (10) after taking the first dose. The threshold and the target range are individual for every patient, the threshold being 10% of the maximum patient’s state (BASE + E_MAX_) and target ranging from 20% to 40% of the maximum value for patient’s state. The algorithm generated all possible schedules, considering all the constraints described in Table 5. The range for doses sizes was specified to [0, 400] for morning dose and [0, 300] for maintenance dose. The schedules considered in this paper used 2 dose step values: 5 mg, to reflect the microtablets used in [29] and 50 mg, to reflect traditionally available levodopa pills. The best schedules generated using the algorithm for the patients are presented in Tables 10 and 11, where score column represents the values calculated using Eq (9).

**Table 10:** Selected medicine intake schedules for each of the patients represented by: The dose time interval (minutes), morning and maintenance dose sizes (mg). The schedules created with the 5 mg dose step. The score column represents the values of the objective function (9).

Patient	Dose interval	Morning dose size	Maintenance dose size	Score
41	90	210	90	30.6
42	90	90	40	83.8
43	90	270	150	27.3
44	90	145	75	25.3
45	100	285	160	15.8
46	90	105	65	42.9
47	90	365	65	29.1
48	90	145	85	56.4
49	90	280	135	22.6
50	90	400	265	40.4

**Table 11:** Selected medicine intake schedules for each of the patients represented by: The dose time interval (minutes), morning and maintenance dose sizes (mg). The schedules created with the 50 mg dose step. The score column represents the values of the objective function (9).

Patient	Dose interval	Morning dose size	Maintenance dose size	Score
41	90	200	100	38.9
42	100	100	50	96.0
43	90	300	150	27.9
44	110	200	100	28.1
45	110	300	200	17.1
46	170	350	200	50.6
47	120	400	100	29.7
48	100	200	100	65.3
49	130	400	250	25.9
50	90	400	300	43.9

Finding these schedules using exhaustive search ensures optimality; however, it takes a lot of computational time (480 s) and can be only performed with many constraints, which leads to excluding some solutions, that might represent better schedules.

### Optimization

To evaluate the patient state prediction model further, optimalization was performed using not only the ML models, but also using the PK/PD model that was used to train the networks, allowing for comparison of their outputs. Two algorithms and a set of constraints from Table 5 were utilized in this evaluation. The following constrains combinations were explored:

- Constraints 1, 2, 3 and 4 with 5 mg and 50 mg doses
- Constraints 1, 2 and 3 with 50 mg doses
- Constraints 1, 2 and 4 with 50 mg doses
- Constraints 1 and 2 with 5 mg and 50 mg doses

Constraint 1 was consistently applied using a time step of 10 minutes, as the impulse models cannot handle doses not taken with a 10-minute step. Constraint 2 ensured that the first dose is taken immediately after wake-up. Constraints 3 and 4 reduced the number of accepted solutions, but simplified the dosing process. Optimization with 5 mg doses was performed in only 2 cases, because the small dose size step allowed for precise dosing and removing a single constraint does not significantly improve the solution. In each of the experiments the (9) objective function was used with an added penalty for every state value below the threshold (10).

Initially, the optimization process using the PK/PD model was performed, to create a benchmark for ML generated models. The first set of constraints (1, 2, 3 and 4) allowed comparing the results with the exhaustive search results—to validate the optimization methods implementations used and their usability for solving this task. Both algorithms successfully found the optimal solution for all patients with the mean time of 4.96 s for differential evolution and 21.9 s for genetic algorithm, demonstrating significantly faster performance than the exhaustive search. The results for other constraints and dose step combinations are presented in Table 12.

**Table 12:** Optimization results for dose step and constraint combinations (based on PK/PD model). The score column represents the values of the objective function (9).

Patient	Score			
	5 mg- 1, 2	50 mg—1, 2	50 mg—1, 2, 3	50 mg—1, 2, 4
41	29.1	32.5	33.8	36.4
42	76.4	92.5	96.0	92.5
43	25.7	25.8	27.9	27.9
44	22.7	27.5	28.1	28.1
45	14.6	16.0	16.3	16.3
46	41.8	48.5	50.5	48.5
47	25.9	28.1	29.7	28.6
48	50.8	63.1	63.1	65.3
49	22.0	24.0	24.6	25.4
50	38.4	39.0	41.7	39.0

Removing constraints for the dose sizes and time intervals between doses led to an improvement in the suggested schedules, the value of the objective function (area outside the target range) decreased on average by 7%. In case of dosing with 5 mg microtablets the result of removing the constraints was not as significant as in case of 50 mg step doses. The ability to precisely set the medicine dose reduces the need for different maintenance dose sizes and different time intervals between doses.

After receiving the expected optimization results for medicine intake schedules using the PK/PD models, optimization was performed using the trained ML model (I-(8)64,64) to evaluate its applicability in optimization of medicine schedules. The achieved schedules slightly differed from the ones achieved using the PK/PD model, usually in the morning dose size. The Tables 13 and 14 present score values for the schedules calculated using the PK/PD model, to be able to compare them with previously received results. The schedules were generated when all 4 constraints were applied.

**Table 13:** Medicine intake schedules for 10 patients acquired through optimization using individual ML models with a 5 mg dose step. The score column represents the values of the objective function (9) calculated using PK/PD models.

Patient	Dose interval	Morning dose size	Maintenance dose size	Score
41	90	180	95	32.4
42	90	90	45	93.6
43	90	230	145	28.6
44	90	145	65	26.2
45	90	220	140	17.0
46	90	110	75	52.5
47	90	105	65	29.8
48	90	110	75	61.2
49	90	245	145	24.0
50	90	335	270	42.4

**Table 14:** Medicine intake schedules for 10 patients acquired through optimization using individual ML models with a 50 mg dose step. The score column represents the values of the objective function (9) calculated using PK/PD models.

Patient	Dose interval	Morning dose size	Maintenance dose size	Score
41	100	200	100	31.2
42	100	100	50	96.0
43	90	250	150	27.8
44	110	200	100	28.3
45	90	250	150	17.8
46	100	200	150	59.0
47	100	350	150	35.0
48	90	150	100	76.2
49	90	250	150	25.3
50	90	300	300	47.0

**Fig 16:**
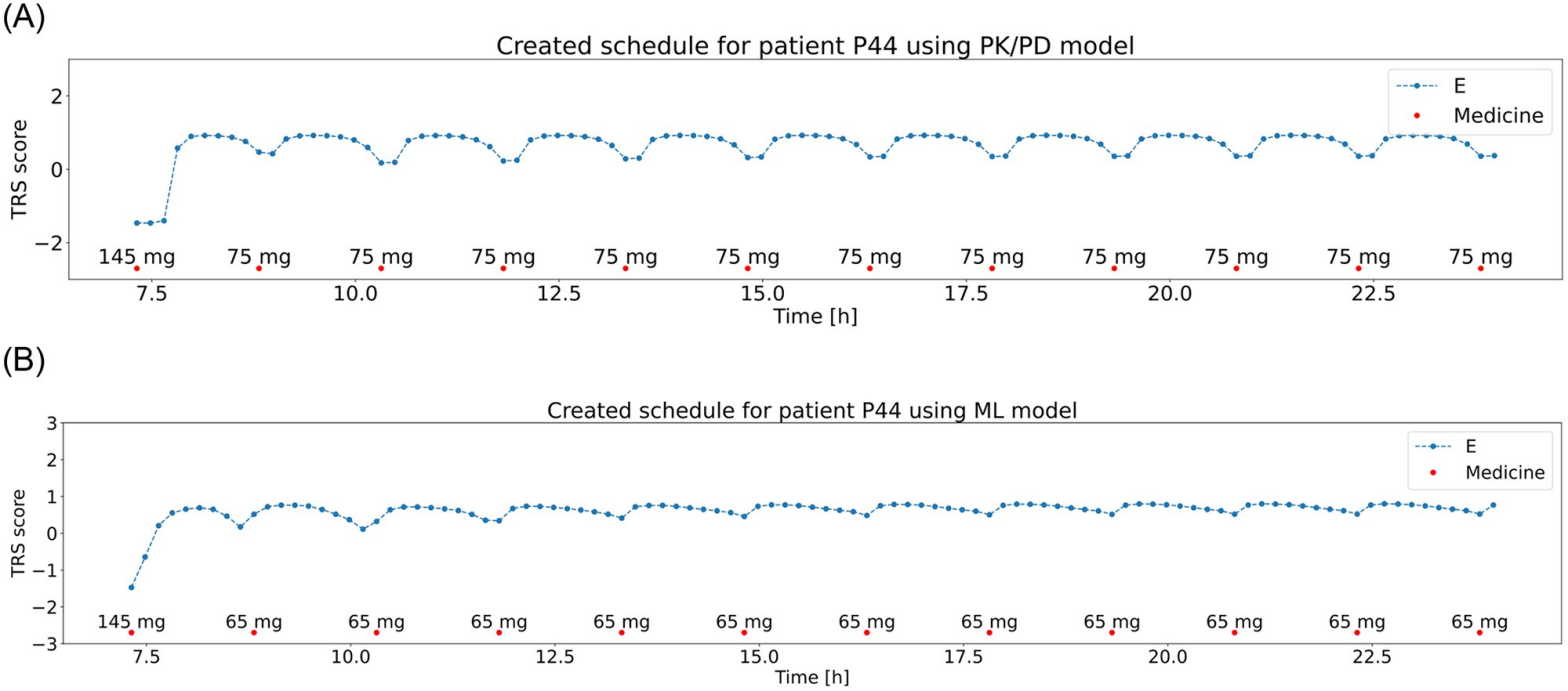
Generated medicine schedule (red dots) using optimization with patient’s states generated with PK/PD model (top) and ML model (bottom) for patient P44 with a dose step 5 mg and patient TRS scores (blue line).

**Fig 17:**
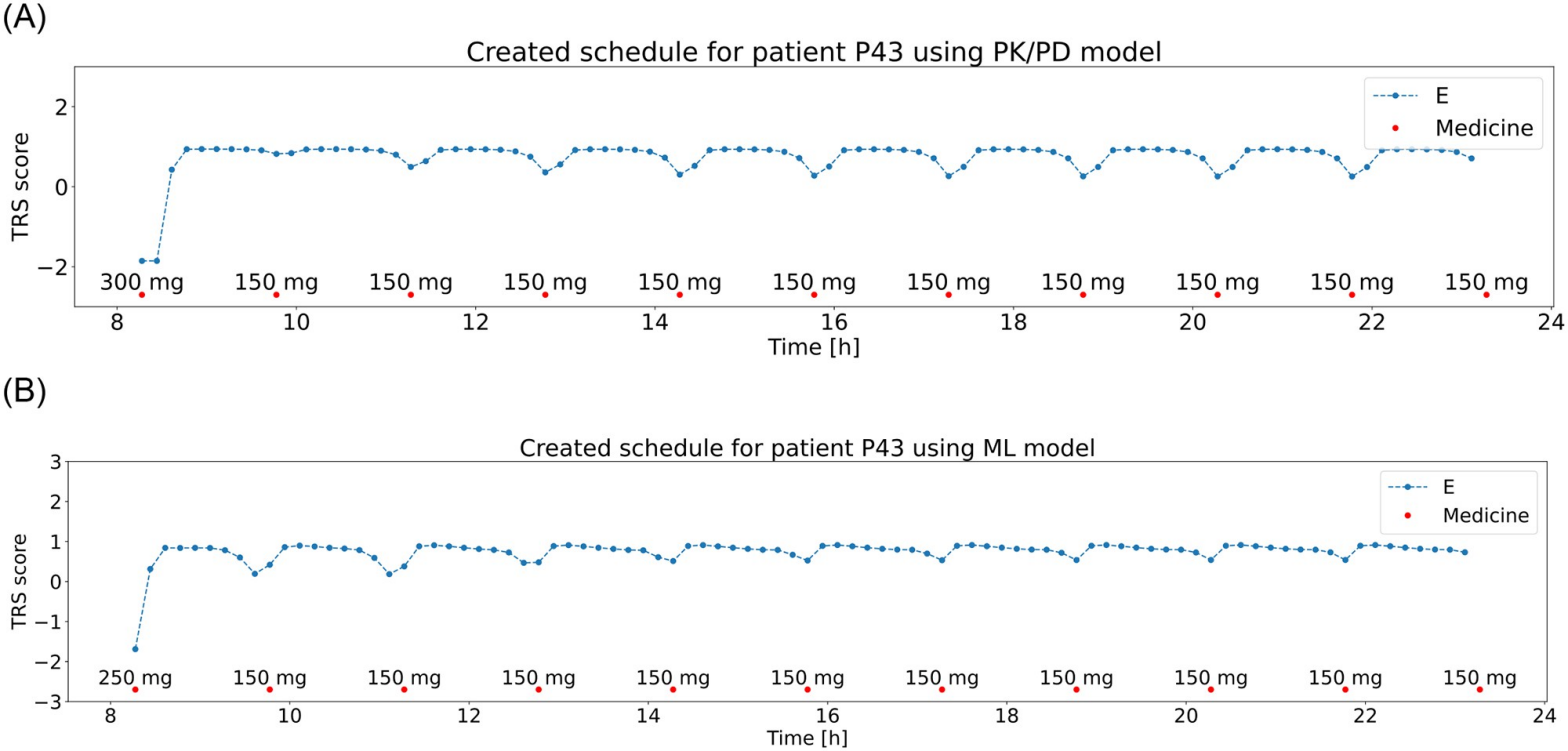
Generated medicine schedule (red dots) using optimization with patient’s states generated with PK/PD model (top) and ML model (bottom) for patient P43 with a dose step 50 mg and patient TRS scores (blue line).

The high objective function values observed in the results acquired with the trained ML model can be caused by the imprecision of the trained model. Due to the small training (2 days) and validation (1 day) sets it was difficult for the model to learn the individual patient responses to medication, considering the fact that the 3 days were generated and included doses that would not typically be taken by the patient. Nevertheless, both the schedules and their evaluation in most cases reflected the sensitivity of the patients to medication, demonstrating the usability of selected ML models. A comparison of schedules generated using the PK/PD and ML models is presented in Fig 16 for a 5 mg dose step and Fig 17 for a 50 mg dose step.

### Reinforcement learning

**Fig 18:**
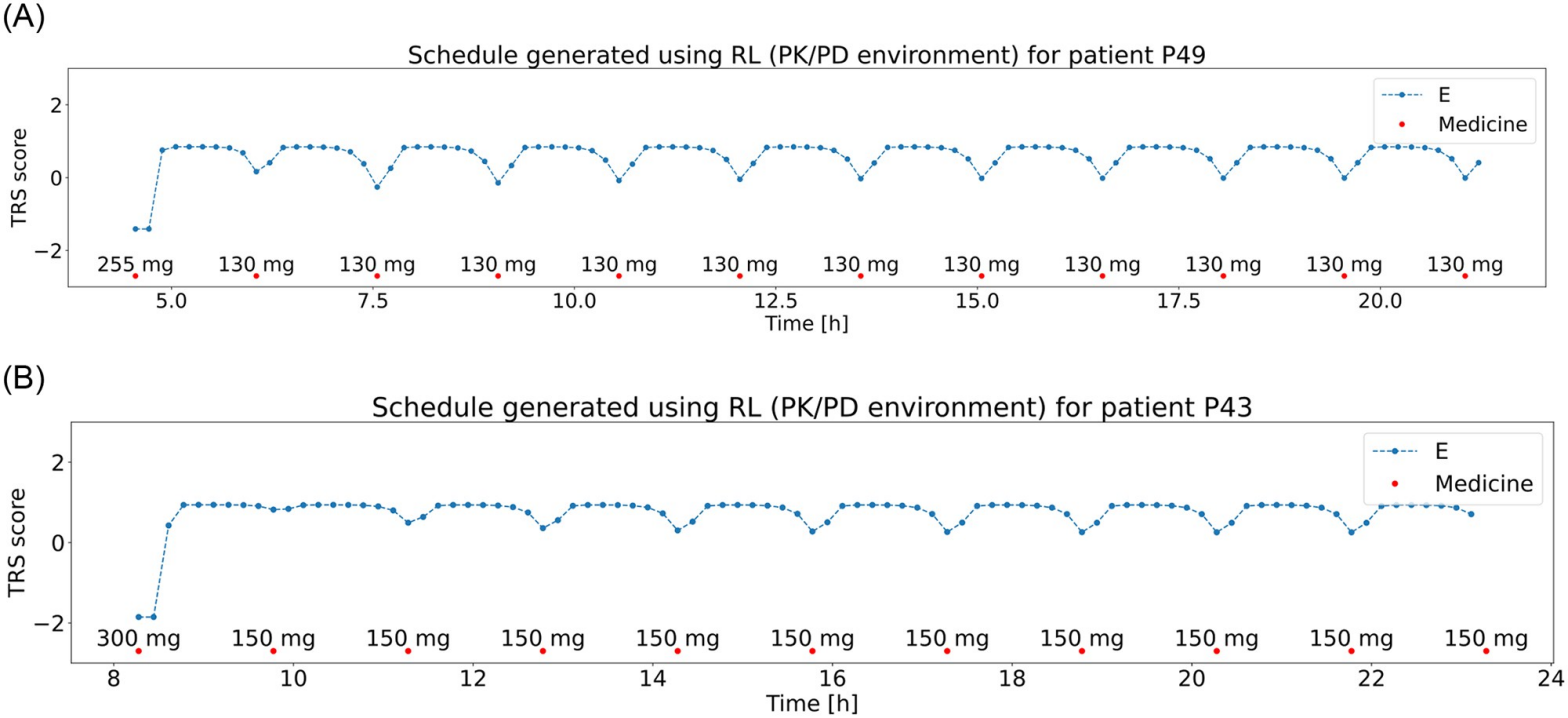
Generated medicine schedule (red dots) using RL with PK/PD environment for patients P49 and P43 with a dose step 5 mg (top) and 50 mg (bottom) with patient TRS scores (blue line).

**Fig 19:**
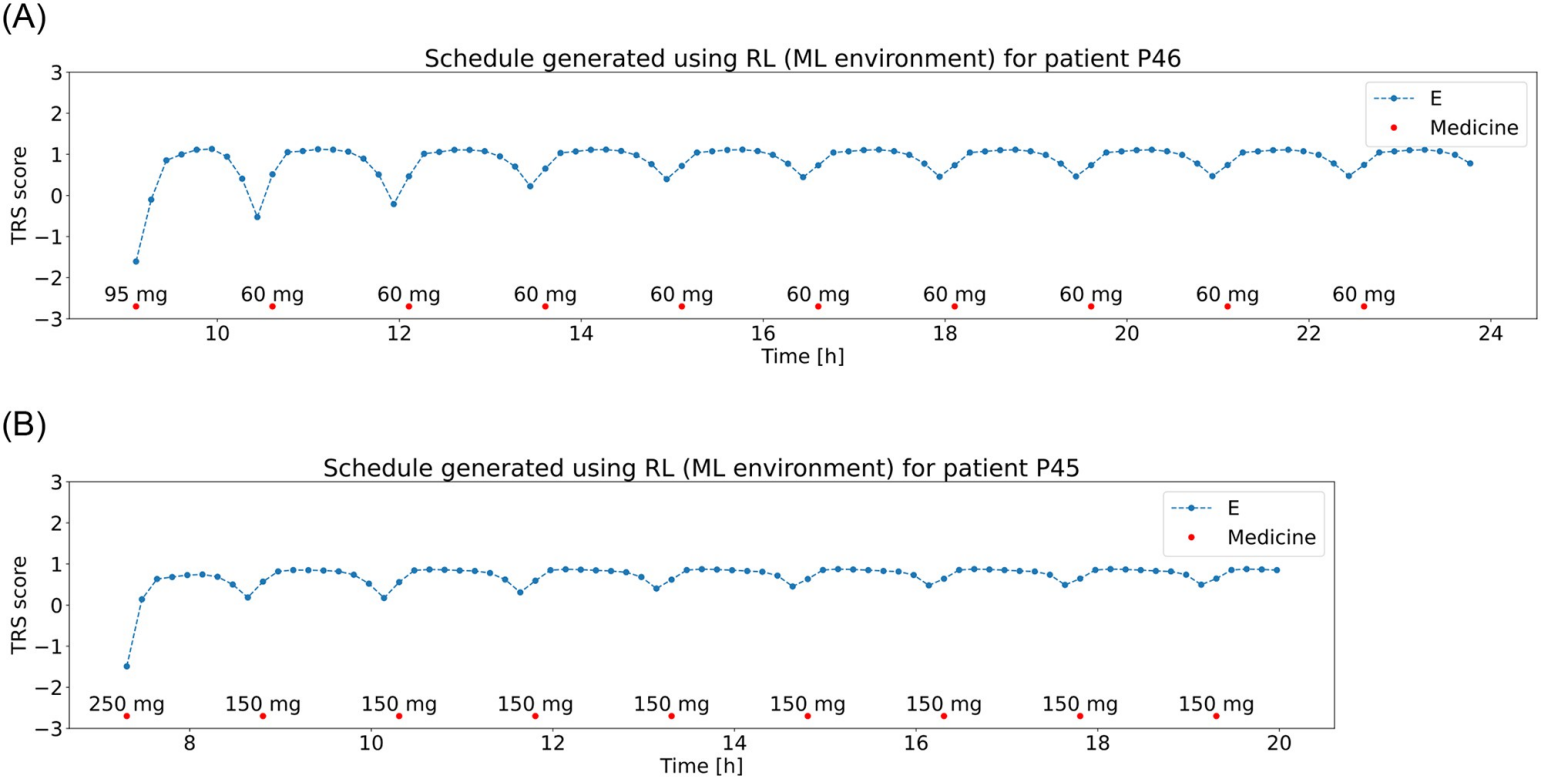
Generated medicine schedule (red dots) using RL with ML environment for patients P46 and P45 with a dose step 5 mg (top) and 50 mg (bottom) with patient TRS scores (blue line).

The reinforcement learning agent was trained using 2 types of constructed environments: first based on PK/PD model and second based on the trained ML model (I-(8)64,64). Two dose steps were used (5 mg and 50 mg) and a minimum time interval of 90 minutes between doses was set to prevent overly frequent doses. The training process was performed with PPO’s default parameters [40]; however, the network architecture was modified. A multilayer perceptron with 2 hidden layers was used, each consisting of 400 neurons for the 5 mg dose step and 200 neurons for the 50 mg dose step. The ReLU activation function was selected and the training was performed for 500 000 timesteps, resulting in similar outcomes to those achieved by classical optimization algorithms. Every 50 000 timesteps a callback was called and if there was no improvement the training process was stopped. After completing, the model was not only able to create a schedule using the initial patient’s state, but also adapt and adjust medication in response to different states. The reward for each schedule was designed to be the negative value of the objective function (9) from the optimalization task. After training, the agents were able to create schedules similar to the ones acquired using previously described optimization methods. The scores (negative rewards) of created schedules are presented in Table 15, while Figs 18 and 19 present examples of generated schedules. For ML the reward was recalculated using the PK/PD model to create comparable data.

**Table 15:** The scores (objective function values) acquired when generating individual schedules for each of the patients using PPO trained agents with PK/PD and ML environments and 2 dose steps—5 mg and 50 mg.

Patient	Score (negative reward)			
	PK/PD—5 mg	PK/PD—50 mg	ML—5 mg	ML—50 mg
41	28.2	38.2	40.5	37.7
42	76.5	86.2	94.9	96.0
43	27.3	27.8	34.8	34.3
44	22.1	28.0	32.3	31.1
45	15.5	16.9	18.9	17.8
46	38.8	46.9	39.6	55.4
47	26.4	28.9	34.4	35.4
48	48.7	63.6	73.1	74.3
49	26.7	25.3	30.0	30.8
50	36.8	38.6	41.3	48.0

The reinforcement learning agent successfully learned the policies that maximized the obtained reward in both the PK/PD and ML environments. Both models enabled creation of schedules that minimized the area outside the target range. However, it should be noted, that in many cases, the results acquired with the ML environment are worse due to the inaccuracy of the trained model.

## Discussion

The study presents results related to three different topics:

- Modelling the PD patient medicine response (levodopa/carbidopa) using machine learning—artificial neural networks.
- Creating medicine intake schedules through optimization, using conventional heuristic optimization methods.
- Creating medicine intake schedules with a trained RL agent.

The success of modelling the individual response to medication using machine and deep learning methods is notable, especially considering the limited number of training samples. These models performed creditably on the validation set and provided promising results in optimization (approaching results of PK/PD model). Acquired performance differed between patients, due to the randomness of dataset generation and individual responses to medication. After performing experiments with two proposed architectures (history and impulse), the advantage of the LSTM architecture was demonstrated, with the best model achieving MSE value of 0.0215 and R^2^ value of 0.965. The history model was able to capture most recent history regarding the taken doses and previous steps, the number of recent considered doses and previous patient steps had to be set at the beginning, even before the training process. However, the impulse model was unable to precisely process medicine doses, since all of times had to be rounded to a 10-minute step. Future experiments considering the ML models will include trials with real patients data and might result in an advantage over PK/PD models, allowing to capture individual traits not considered in the models and to accept additional information regarding the patient’s food consumption and physical activity performed during the day.

The optimization process for creating daily schedules was inspired by research about building sensor-based algorithmic dosing suggestions [29]. The research presented in this paper goes a step further and uses optimization methods for creating such schedules which results in better performance and gives more flexibility when considering the solution space. Using the same objective function (as in the source article) allows to verify that such an approach can provide better results. The framework can be used to generate a general medicine schedule for the patient and equipped with a mobile device and sensors can be capable of adapting to the patient’s current condition. However, recalculating the optimization for the remaining part of the day may be necessary to improve the patient’s quality of life.

Creating medicine intake schedules using reinforcement learning has provided good results with both environments: PK/PD and ML model. Defining the reward using the previously used objective function allowed to compare the results with the heuristic optimization algorithms results. Although the schedules were similar, RL provided slightly worse results in some cases. However, RL offers the advantage of real-time adaptation without any additional training or time-consuming computations, allowing for step-by-step adjustments based on the patient’s current state.

Presented methods for creating medicine intake schedules have been used with both—the PK/PD models and trained individual ML models. The ML models provided slightly worse results compared to PK/PD models due to the small size and diversity of the training set. Future research will include trials with more training data—more than 3 days per patient, collected through sensor examinations with a mobile application and a sensor armband.

The experiments in this paper were performed using generated data that closely reflected real conditions, following a validated generation method [28]. Data collection was limited to a maximum of 3 days, resembling typical hospital visits, with measurements recorded at 10-minute intervals, consistent with the original study. The optimization process was performed with multiply variants of constraints to enable comparisons with previously published results on medicine schedules generation.

The proposed method might raise questions about its applicability, as it requires frequent data collection (to evaluate the patient’s condition) and patient’s cooperation. However, once the model is trained, examinations can be less frequent, to verify that the current schedule is sufficient and no adjustments are needed. In this study the 10-minute intervals have been chosen (to compare results with cited publications); however, the framework allows for customization of the time intervals. Continuous evaluation solution are also being developed—can perform the evaluation of patient’s state in the background [7]. Once they allow to accurately predict the current condition they might reduce the need for performing examinations with predefined intervals.

Creating medicine intake schedules and adapting them to the patient’s needs with machine learning is a sensitive topic that demands precision, as errors in scheduling may have adverse consequences. Patient may experience not only PD symptoms that degrade their quality of life but also levodopa induced dyskinesias that make it hard for the patient to complete daily activities. To minimize the risk of inadequate medicine dosing suggestions, all proposed schedules should verified by the clinician before application. Future development of the framework will include safeguards that will verify the solution and alert the clinician if the proposed schedule is not sufficient, enabling necessary steps to provide successful treatment.

## Conclusions

The paper demonstrates the successful application of supervised and reinforcement machine learning along with conventional optimization methods in creating medicine intake schedules for PD patients. Achieving the aim of building a framework, we have presented a robust solution that, pending further validation on patient data, can improve the workflow of neurologists by offering an objective and user-friendly dosing system.

The findings indicate that the approach not only meets the objective of minimizing errors in therapy but also holds the promise of improving the patient’s quality of life. The introduced method reduces the time and complexity involved in creating individualized medicine schedules, thereby potentially improving both treatment results and overall patient well-being.

In conclusion, the solution presented is ready for real-world application but requires further verification and training on larger datasets from real patients. Once fully validated, the method has considerable potential to modify the treatment scheduling for Parkinson’s disease, adding significant value to modern medical practice.

## Supporting information

S1 File Simulated data for 50 PD patients. This CSV file includes detailed simulated medication and state data for 50 PD patients, based on the PK/PD models described in the paper.
(ZIP)

S2 File Python source code of the framework. This supplementary file contains the complete Python source code for training the machine learning models to predict patient medication responses and for generating optimized medication schedules.
(ZIP)
